# Demographic history and local adaptation of *Myripnois dioica* (Asteraceae) provide insight on plant evolution in northern China flora

**DOI:** 10.1002/ece3.7628

**Published:** 2021-05-17

**Authors:** Nan Lin, Jacob B. Landis, Yanxia Sun, Xianhan Huang, Xu Zhang, Qun Liu, Huajie Zhang, Hang Sun, Hengchang Wang, Tao Deng

**Affiliations:** ^1^ CAS Key Laboratory for Plant Diversity and Biogeography of East Asia Kunming Institute of Botany Chinese Academy of Sciences Kunming China; ^2^ CAS Key Laboratory of Plant Germplasm Enhancement and Specialty Agriculture Wuhan Botanical Garden Chinese Academy of Sciences Wuhan China; ^3^ College of Life Science Henan Agricultural University Zhengzhou China; ^4^ School of Integrative Plant Science Section of Plant Biology and the L.H. Bailey Hortorium Cornell University Ithaca NY USA; ^5^ Center of Conservation Biology Core Botanical Gardens Chinese Academy of Sciences Wuhan China; ^6^ School of Life Sciences Yunnan Normal University Kunming China

**Keywords:** demographic history, effective population size, genomic variations, local adaption, *Myripnois dioica*, RAD‐seq

## Abstract

The flora of northern China forms the main part of the Sino‐Japanese floristic region and is located in a south–north vegetative transect in East Asia. Phylogeographic studies have demonstrated that an arid belt in this region has promoted divergence of plants in East Asia. However, little is known about how plants that are restricted to the arid belt of flora in northern China respond to climatic oscillation and environmental change. Here, we used genomic‐level data of *Myripnois dioica* across its distribution as a representative of northern China flora to reconstruct plant demographic history, examine local adaptation related to environmental disequilibrium, and investigate the factors related to effective population size change. Our results indicate *M. dioica* originated from the northern area and expanded to the southern area, with the Taihang Mountains serving as a physical barrier promoting population divergence. Genome‐wide evidence found strong correlation between genomic variation and environmental factors, specifically signatures associated with local adaptation to drought stress in heterogeneous environments. Multiple linear regression analyses revealed joint effects of population age, mean temperature of coldest quarter, and precipitation of wettest month on effective population size (*Ne*). Our current study uses *M. dioica* as a case for providing new insights into the evolutionary history and local adaptation of northern China flora and provides qualitative strategies for plant conservation.

## Graphical abstract

Our current study uses *Myripnois dioica* as a case for providing new insights into the evolutionary history and local adaptation of northern China flora and provides qualitative strategies for plant conservation which have been generally overshadowed.

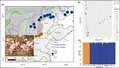

## 1 INTRODUCTION

The Sino‐Japanese floristic region (SJFR) is one of the oldest and richest area of temperate floral elements in the North Hemisphere, and the northern China flora composes the main part of the SJFR (Chen et al., 2018; Gao et al., 2003; Lu et al., 2018; Wu & Wu, 1996). Northern China has served as an important region composing the north–south vegetation transects from tropical to cold forests and taiga, and paleovegetation evidence suggests that the current components of the northern China flora are mainly derived from the Neogene–Quaternary period (Gao et al., 2003; Manchester et al., 2009; Wang, 1999). Covering a relatively wide geographic location, along with past climatic oscillations and geological changes, a high number of species are found in northern China (Wu et al., 2005; Wu & Wu, 1996; Yu et al., 2000), of which are an abundance of endemic species, such as *Xanthoceras sorbifolium* Bunge (Sapindaceae), *Opisthopappus taihangensis* (Ling) Shih (Compositae), and *Taihangia rupestris* Yu et Li (Rosaceae) (Wang, 1999). Genetic evidence has suggested that plants in the northern China region contracted to southern glacial refugia during the last glacial maxima (LGM) and then expanded northwards during postglacial migration. This hypothesis is supported in present‐day phylogroups with northern region populations originating from recent range expansions of a southern refugium, which may present founder and bottleneck effects with reduced genetic diversity within and between populations (Cao et al., 2015; Chen et al., 2008; Hao et al., 2018; Tang et al., 2018; Tian et al., 2009). Alternative hypotheses include the remnants of multiple geographically isolated refugia occurring in northern China causing population differentiation of many plant species in the region (Hou et al., 2020; Wang et al., 2017; Ye et al., 2019). Studies addressing both hypotheses have predominately been limited to using several loci and almost exclusively conifer species, which inadequately address the questions. Thus, genome‐level data and plants with shorter life cycles can help to better demonstrate the evolution of plants making up the northern China flora.

Physical barriers containing multiple mountains and drainage systems have acted on species differentiation in northern China, such as the Taihang Mountains promoting intraspecies divergence (Hou et al., 2014; Ye et al., 2018). In addition, the uplift of the Qinghai–Tibetan Plateau (QTP) has aggravated monsoon intensity and enhanced aridity in the Asian interior, which profoundly influenced the climate of northern China (Guo et al., 2008; Li et al., 2001; Shi et al., 1999). With the formation of an east–west arid belt, this ecological barrier played a significant role in plant divergence and can be tested with phylogeographic studies (Bai et al., 2016; Guo et al., 2008). Thus, northern China is a model region for studying how these intricate factors together influence the evolution of plants.

In response to selective pressures from environmental heterogeneity, species undergo local adaptation that can be identified using population genetic analyses based on genome–environment association data (Joost et al., 2007; Li et al., 2017). Many cases based on nonmodel plant species have illustrated environmental factors including soil, temperature, and precipitation have played important roles in driving differentiation in locally adaptive species (Chao et al., 2020; De Kort et al., 2014; Jones et al., 2013). The regional climate impacting the local flora of northern China is arid with small quantities of precipitation that may be the major environmental factors effecting adaptive evolution. Previous studies in *Phyteuma hemisphaericum* L. (Campanulaceae), *Campanula barbata* L. (Campanulaceae), and *Carex sempervirens* Vill. (Cyperaceae) (Basu et al., 2016; Jones et al., 2013; Manel et al., 2012) identified genetic variation that is highly related to precipitation factors, emphasizing local adaptation within species to cope with drier climatic conditions. However, few environmental–genetic studies have been reported for the flora of northern China, and the link to whether/how the evolution of plant populations is driven by adaptation to drought in this region has not fully been investigated. In this study, we use genome‐wide data to find correlations between loci and ecological factors based on a representative species to explore adaptive evolution pattern of regional vegetation in the northern China flora.

**FIGURE 1:**
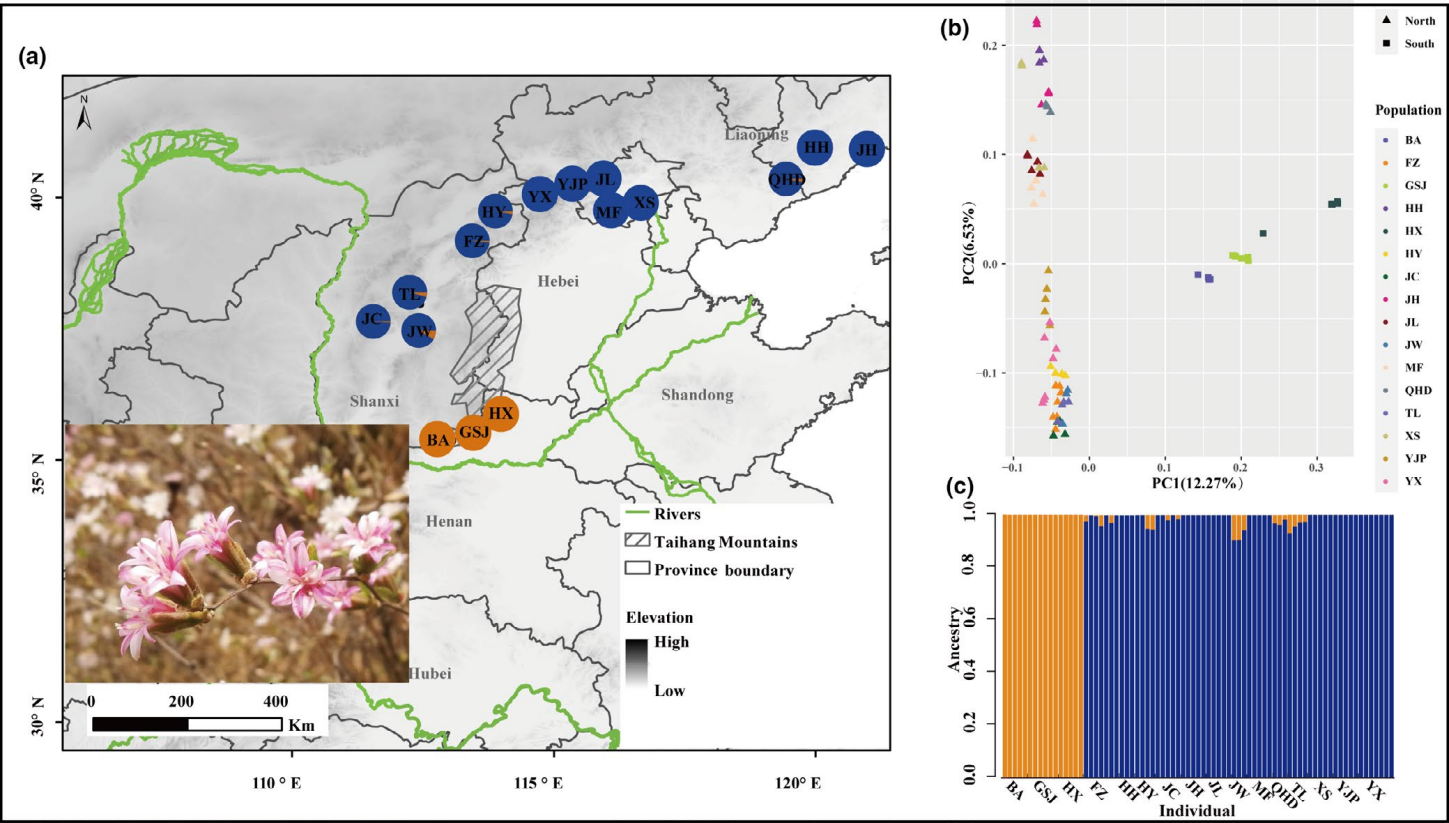
(a) Geographical distribution of two genomic groups of *Myripnois dioica* based on ADMIXTURE; populations are color‐coded corresponding to different groups. (b) Plot of individuals of *M. dioica* along principal component analysis (PCA) scores of genetic variations based on the analysis of 22,868 SNPs; the triangles and dots are consistent with south and north groups. (b) Plots of posterior probabilities for individuals of *M. dioica* assigned to *K* genetic clusters from Admixture analyses for *K* = 2. Population names listed along the bottom of the plot and the south and north groups are delimited by yellow and blue color

*Myripnois dioica* Bunge is a temperate, deciduous shrub species ranging from 35°N to 45°N (Figure 1), geographically overlapping the arid belt. The species belongs to the daisy family and is wind‐dispersed with seeds having long pappi, but is distinguishable from other closely related species by its shrub life form (Gao & Nicholas, 2011). There is little known about this long‐neglected species except for its ornamental value (Xie & Zhang, 2012; Xu et al., 2007). The species is widely distributed along the Taihang Mountains in habitats of uneven altitude from 200 m a. s. l. to 1,500 m a. s. l. and can survive in dry, cold, and rocky conditions (Gao & Nicholas, 2011; Xie & Zhang, 2012). Since *M. dioica* is one of the dominate components of shrub community in northern China, the species has undergone complicated geographic and climatic changes together with extreme ecological adaption. Thus, this species is an ideal system to investigate the joint effects of Pleistocene climatic oscillations, biogeographic barriers, and patterns of ecological adaption in the flora of northern China.

Given the complex factors contributing to the evolution of *M. dioica* in northern China, efforts in identifying and quantifying important factors will undoubtedly lead to better conservation strategies, providing an evolutionary diversity reference for the framework of conservation genetics (Jarzyna & Jetz, 2016; Moritz & Potter, 2013). Phylogeography has been shown to be useful in plant conservation by investigating historical and evolutionary questions utilizing genetic structure, spatial, and demographic perspectives (Médail & Baumel, 2018). Studies in plants have also suggested that both expansion history and abiotic climate directly impact effective population size variation (Braasch et al., 2019; Micheletti & Storfer, 2015). In this case, inherited mechanisms for how specific historical and climatic factors affect species evolution and population size changes could protect plant species from extinction under disturbances by human encroachment and global climate change (Pauls et al., 2013; Polechová & Storch, 2008; Pritchard et al., 2000).

Genome‐wide data derived from restriction enzyme‐associated DNA sequencing (RAD‐seq) have been effective and well‐suited for phylogeographic studies and investigating adaptive evolution (Haselhorst et al., 2019; Warschefsky & von Wettberg, 2019). Large numbers of markers can be obtained from RAD‐seq to identify evolutionary patterns that are not easily visible in traditional analyses based limited loci. In the current study, we employed *M. dioica* as a representative member of the flora of northern China to identify phylogeographic patterns and processes impacting this species. Our goals are to (a) investigate demographic history of *M. dioica*; (b) explore local adaptation of *M. dioica* to dry environmental conditions in northern China; (c) address specific mechanism for potential historical and environmental factors related to changes in effective population size of *M. dioica*.

## 2 MATERIALS AND METHODS

### 2.1 Sampling, DNA extraction, and RAD sequencing

We sampled 77 individuals from 16 populations spanning the geographic range of *M. dioica*. Fresh leaves from each individual were dried in silica gel. Total genomic DNA was extracted using a modified cetyltrimethylammonium bromide (CTAB) method (Doyle et al., 1996). The quality of DNA was visualized on a 1% agarose gel and quantified on a Qubit^®^ 2.0 fluorometer. Genomic DNA was digested with the restriction enzyme EcoRI, and restriction site‐associated DNA library construction and sequencing were performed by the Beijing Genomics Institute BGI (Wuhan, China) with paired‐end sequencing on an Illumina HiSeq 2000 platform. The raw data were deposited in the NCBI Short Read Archive (SRA) (Bioproject No. PRJNA607823).

### 2.2 Bioinformatics pipeline for SNP calling

All 77 individuals from 16 populations were used for de novo SNP calling (Table S1 and Figure 1). Only the forward reads of the paired ends were used for analyses due to low coverage of the reverse reads. Reads were filtered for quality and demultiplexed using process_radtags with default parameter in Stacks 2.0 (Catchen et al., 2013). We then used the denovo_map.pl wrapper to identify SNPs by clustering reads with a minimum number of four raw reads and allowing two mismatches between loci. Given the limited number of individuals within each population, the populations command was used to filter loci so that polymorphic loci in at least 50% of the individuals and twelve populations were retained. We further removed loci with minor allele frequencies <0.05 and only included the first SNP per locus in the final analysis to avoid linkage bias. Finally, a maximum observed heterozygosity was set to 0.5. PGDSPIDER 2.1.1.5 and VCFtools 0.1.15 were used to generate input files for downstream analyses (Danecek et al., 2011; Lischer & Excoffier, 2012).

### 2.3 Summary statistics and population structure

We used the populations command of Stacks to estimate nucleotide diversity (*π*), observed heterozygosity (*H*_O_), expected heterozygosity (*H*_E_), and fixation index (*F*_IS_) for each population. We performed pairwise population *F*_ST_ values using an analysis of molecular variance (AMOVA) with 1,000 permutations as implemented in ARLEQUIN 3.0 (Excoffier et al., 2005). Population structure analyses were performed to infer the most likely number of ancestral populations using ADMIXTURE 1.23 (Alexander et al., 2009) by determining the optimal partitioning of the populations according to cross‐validation error value of clusters 1–10. A principal component analysis (PCA) using the genome‐wide complex trait analysis (GCTA) was used to explore the genetic structure and visualized in R (Pluess et al., 2016; RC, 2013). RAxML v8.2.10 (Stamatakis, 2014) was used to construct a maximum‐likelihood (ML) phylogeny of the 16 populations from unlinked SNPs using the ascertainment bias correction for the GTRGAMMA model (‐m ASC_GTRGAMMA, –asc‐corr = lewis) with 1,000 bootstrap replicates.

### 2.4 Demographic history and species distribution modeling

Effective population size (*Ne*) for each population was estimated using the molecular co‐ancestry method implemented in NEESTIMATOR 2.01 (Do et al., 2014). To explore the historical demography of *M. dioica*, we employed DIYABC v. 2.0 software based on an approximate Bayesian computation algorithm (ABC) (Cornuet et al., 2014). The 16 populations samples were divided into two groups (group N and group S) based on genetic structure, PCA, and phylogenetic relationships of *M. dioica*, and we only used a single SNP per locus and neutral SNPs and without missing data for the ABC simulation. Most populations are distributed in the northern Taihang Mountains, and we first tested four possible divergence scenarios to estimate whether *M. dioica* originated from the northern region (Figure S1). We employed a uniform prior probability and selected all summary statistics to generate a reference table based on 4 × 10^6^ simulated datasets with 1% of the simulated datasets closest to the observed data to estimate the relative posterior probabilities for all scenarios. Based on our field observations and closely related species (Zhao & Gong, 2015), we set a conservative estimate for generation time to 5 years to estimate *M. dioica* demographic history. Additionally, we investigated demographic scenarios of changes in population size in two groups of *M. dioica*. Five models of population size changes were used with the same parameter settings strategy as above to choose the best fit demographic scenario and parameter estimation (Figure S1; Fan et al., 2018; Wang et al., 2016). Gene flow patterns among different populations were analyzed with setting the number of migration events from 1 to 3 in the TreeMix 1.11 software (Pickrell & Pritchard, 2012). To further test the effects of glaciation on *M. dioica*, species distribution modeling was generated with MAXENT 3.2 (Phillips et al., 2006) using occurrence data gathered from field collections and herbarium specimens. After eliminating highly correlated variables, six bioclimatic variables from WorldClim (http://www.worldclim.org) (Hijmans et al., 2005) were used as environmental predictors for species distribution modeling (SDM). The following two layers from the Model for the Community Climate System Model (CCSM, (Hasumi & Emori, 2004)) were employed for distribution modeling: the current layer and the last glacial maximum (LGM: *c*. 21 ka) layer. For evaluation, models were calibrated on 75% of the data and evaluated on the remaining 25% using area under the curve (AUC) and replicated 10 times.

### 2.5 Genomic variations and outliers related to climate‐driven local adaptation

To assess the correlation between geographic distance and genetic distance, we conducted isolation‐by‐distance (IBD) analyses using ARLEQUIN (Excoffier et al., 2005). We also tested the contribution of environmental differences to population differentiation (IBE) by comparing pairwise *F*_ST_ values and environmental distances among populations using a Mantel test in GenAlEx 6.3 with 1,000 permutations (Peakall & Smouse, 2006). A total of 19 bioclimatic variables for all populations under current conditions were obtained from of WorldClim at 30‐arc seconds resolution (1960–1990, http://www.world clim.org). We first conducted a principal component analysis (PCA) to obtain the first two principal components of the 19 bioclimatic variables (Clim_PC1 and Clim_PC2), and these values were used as points in two dimensions to calculate a pairwise distance matrix (Pluess et al., 2016). To evaluate genomic variation and its association with geographic and environmental factors, redundancy analyses (RDA) were used to partition the variance of genomic variation explained by variables (Peres‐Neto et al., 2006) as implemented in the VEGAN 2.5.7 package in R (Oksanen, 2020). Our initial model was as follows: Y (individual genotype) ~ Latitude + Longitude + Clim_PC1 + Clim_PC2, and we also tested the significance of the estimated variance explained by all variables and separate variables with 999 permutations. Furthermore, a gradient forest (GF) analysis was performed using the R package gradientForest 0.1‐17 (Ellis et al., 2012), which investigated the relationships between genomic variables and the six irrelevant bioclimatic variables.

To detect signatures of natural selection, we first used BayeScan 2.10 following the Bayesian‐likelihood method of reversible‐jump MCMC, which is based on a Dirichlet distribution decomposing locus‐population *F_ST_* coefficients into a population‐specific component (beta) (Foll & Gaggiotti, 2008). Moreover, a burn‐in of 50,000 iterations followed by 50,000 iterations was used for estimation using a thinning interval of 20. We then used BayPass 2.1 to identify climatic associations with all SNPs (Gautier, 2015) and to detect signature of adaptive selection associated with population‐specific covariates (Clim_PC1 and Clim_PC2). Consensus sequences of outlier loci were aligned to the available genome of sunflower, *Helianthus* *annuus* L. (Badouin et al., 2017) for annotation.

### 2.6 Optimization linear modeling of effective population size, expansion dynamics, and climatic factors

The current distribution of *M. dioica* in northern China may suggest the important role of climate variation or expansion history in demographic performance. We further tested for the influence of the demographic history and climate on effective population size (*Ne*) among populations. We divided the 19 climate factors into two types associated with energy and moisture factors and used the subsequent principal component (PC) axes to describe climatic variation. We estimated the date of colonization for each population (hereafter named population age). Bayesian reconstructions were performed for 16 *M. dioica* populations using MrBayes v3.2.6 under GTR+I+G model based on four makers (*trnL‐F*, *matK*, *ndhF*, and *psbA‐trnH*; accession number: MW380873–MW380901, MW464868–MW464895) (Ronquist & Huelsenbeck, 2003), with *Pertya rigidula* (Miq.) Makino selected as an outgroup based on Funk et al. (2014). In order to estimate divergence time among populations, molecular dating was performed in BEAST 1.6 using uncorrelated log‐normal (UCLN) relaxed‐clock model (Drummond & Rambaut, 2007). The root node was constrained using a normal prior distribution using secondary calibration based on the results of Funk et al. (2014). Sister populations sharing one node were identified as having a consistent colonization history. To quantify the contributions of both population age and climatic environment to variation in *N_e_* for populations, we used a general linear model using Akaike information criterion in the R package STATS 3.0.2, with *N_e_* as the dependent variable of population age, climatic factors, and their interactions as explanatory variables (RC, 2013).

## 3 RESULTS

### 3.1 RAD processing

After demultiplexing, and filtering low‐quality reads of 16 populations (Table S1 and Figure 1), we obtained an average of ~1.6 million reads per sample with detailed information presented in Table S2. Our Stacks pipeline with subsequent filtering generated 22,868 SNPs within *M. dioica*.

### 3.2 Population structure

**FIGURE 2:**
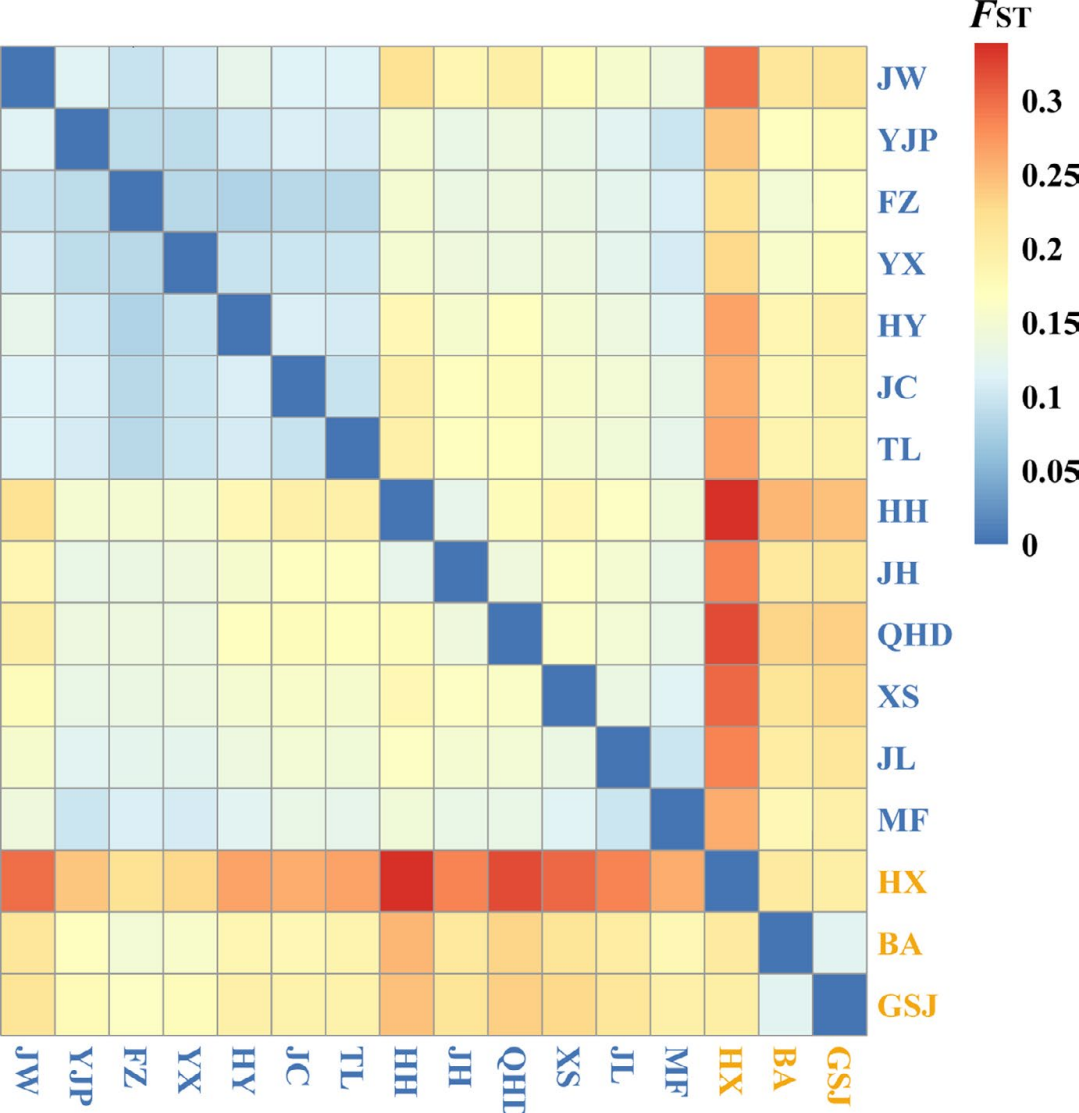
Heatmap of pairwise values among 16 populations of *Myripnois dioica* based on 22,868 SNPs. Populations from the south and north groups are labeled by yellow and blue color

Based on the 22,868 SNPs, the average within‐population nucleotide diversity (*π*) was 0.0347 ranging from 0.0303 to 0.0384 in populations JL and JW, respectively (Table 1). Our Pearson's product–moment correlation revealed that there was no correlation between the number of individuals and nucleotide diversity (*r* = −0.21, *p* = 0.44). Meanwhile, average expected heterozygosity (*H*_E_) and observed heterozygosity (*H*_O_) across all populations were 0.1433 and 0.1915 (Table 1). The average level of differentiation between populations as reflected by pairwise *F*_ST_ values was 0.081 (FZ vs. HY) to 0.339 (HH vs. HX) indicating uneven genetic differentiation (Figure 2, Table S3). Analysis of all SNPs with admixture, ML phylogeny, and PCA revealed a well‐supported split at *K* = 2 corresponding to geography, and the populations included in the southern group (BA, GSJ, and HX; hereafter group S) and northern group (the rest populations; hereafter group N) were separated by the Taihang Mountains (Figures 1 and S2).

**TABLE 1:** Detailed summary statistics per population based on 22,868 restriction site‐associated DNA sequencing SNPs in *Myripnois dioica*

Population	*N*	Pi	*H*_O_	*H*_E_	*F*_IS_	*N*_e_ (95% CI)	Population age (Ma)
BA	5	0.0346	0.14	0.10	−0.03	3.3 (3.2–3.5)	2.71
FZ	7	0.0348	0.21	0.17	−0.05	8.0 (7.6–8.5)	1.38
GSJ	6	0.0346	0.18	0.13	−0.07	1.9 (1.8–1.9)	1.02
HH	3	0.0380	0.22	0.15	−0.05	Infinite	0.41
HX	5	0.0351	0.18	0.10	−0.11	4.7 (3.9–4.3)	1.02
HY	4	0.0369	0.20	0.16	−0.03	15.6 (14.1–17.1)	0.60
JC	5	0.0378	0.19	0.15	−0.05	7.0 (6.6–7.3)	1.17
JH	5	0.0342	0.21	0.16	−0.06	3.3 (3.2–3.4)	1.16
JL	5	0.0303	0.22	0.17	−0.06	4.6 (4.5–4.7)	1.16
JW	3	0.0384	0.22	0.15	−0.04	10.6 (9.7–11.5)	0.41
MF	5	0.0329	0.22	0.18	−0.05	6.6 (6.3–6.9)	3.63
QHD	3	0.0341	0.20	0.16	−0.01	Infinite	0.76
TL	4	0.0356	0.21	0.16	−0.05	12.7 (11.7–13.8)	1.49
XS	5	0.0310	0.24	0.17	−0.09	4.5 (4.4–4.7)	2.38
YJP	5	0.0320	0.22	0.17	−0.04	5.6 (5.4–5.8)	0.60
YX	7	0.0344	0.21	0.17	−0.06	3.4 (3.3–3.5)	0.97

Note

*N*, the number of individuals analyzed; Pi, nucleoid diversity; *H*_O_, observed heterozygosity; *H*_E_, expected heterozygosity; *F*_IS_, fixation index; *N*_E_ (95% CI), effective population size estimates with 95% confidence intervals; population age, divergence time of each population.

### 3.3 Demographic history and species distribution modeling

**FIGURE 3:**
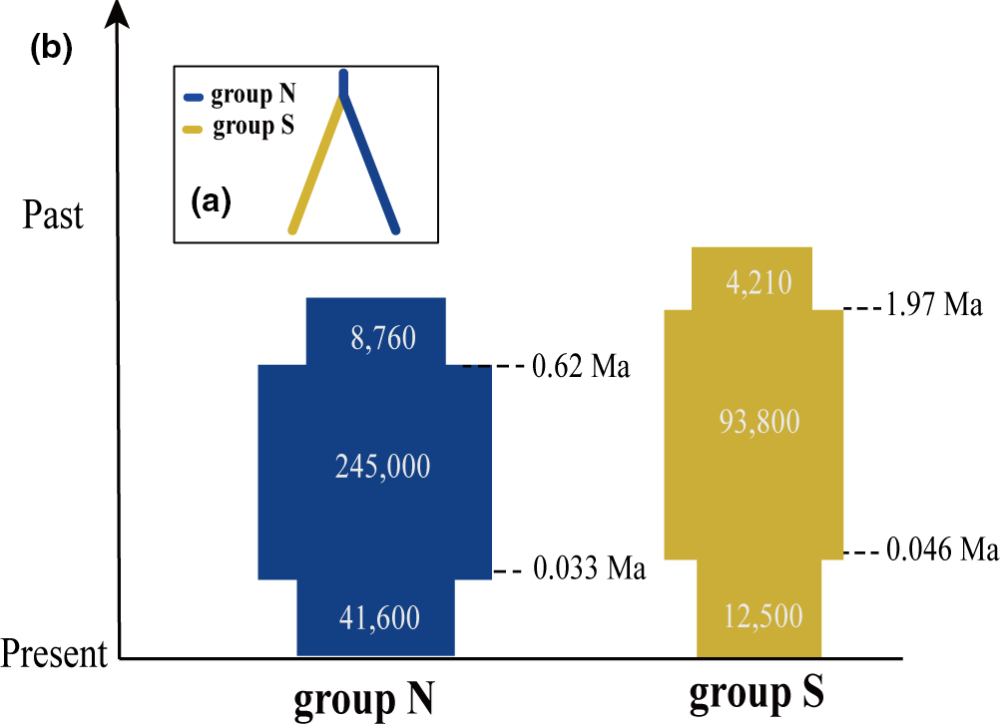
(a) The best ABC divergence model for *Myripnois dioica* based on diy‐abc analyses; (b) Demographic history of the two lineages under the best‐fitting ABC models. Times of population size changes are indicated by horizontal dashed lines

Among populations, *N_e_* estimates ranged from 1.9 to 15.6 with the lowest and highest *N_e_* detected in population GSJ and HY, respectively (Table 1). In southern populations, *N_e_* estimates ranged from 1.9 to 4.7 (populations GSJ and HX), while in northern populations the values ranged from 3.3 to 15.6 (populations JH and HY). DIYABC estimations of the divergence history of *M. dioica* indicated that the scenario 3 had the higher posterior probability (posterior probabilities = 0.805, 95% CI: 0.579–1.000) (Figure S1 and Table S4). For demographic history, the best fit scenario for both group N and group S was Scenario 4 of *M. dioica* (Figures 3, S1 and Table S4). Group N was found to be the ancestral population and started to expand its distribution at *c*. 0.62 Ma (95% CI: 0.14–4.54 Ma), followed by a bottleneck at *c*. 0.033 Ma (95% CI: 0.003–0.094 Ma). Group S diverged from the ancestral population at *c*. 2.37 Ma (95% CI: 2.25–4.11 Ma), followed by expansion at 1.97 Ma (95% CI: 1.41–2.94 Ma) and a bottleneck at 0.046 Ma (95% CI: 0.029–0.097 Ma). Using TreeMix to infer migration patterns among different populations, we did not find significant gene flow between populations (not shown). Species distribution modeling revealed the optimal habitat for *M. dioica* has changed minimally during the LGM (Figure S3), and there are two main habitable areas located at 40°N and 37.5°N, respectively.

### 3.4 Genomic variation and outliers related to local adaptation

**FIGURE 4:**
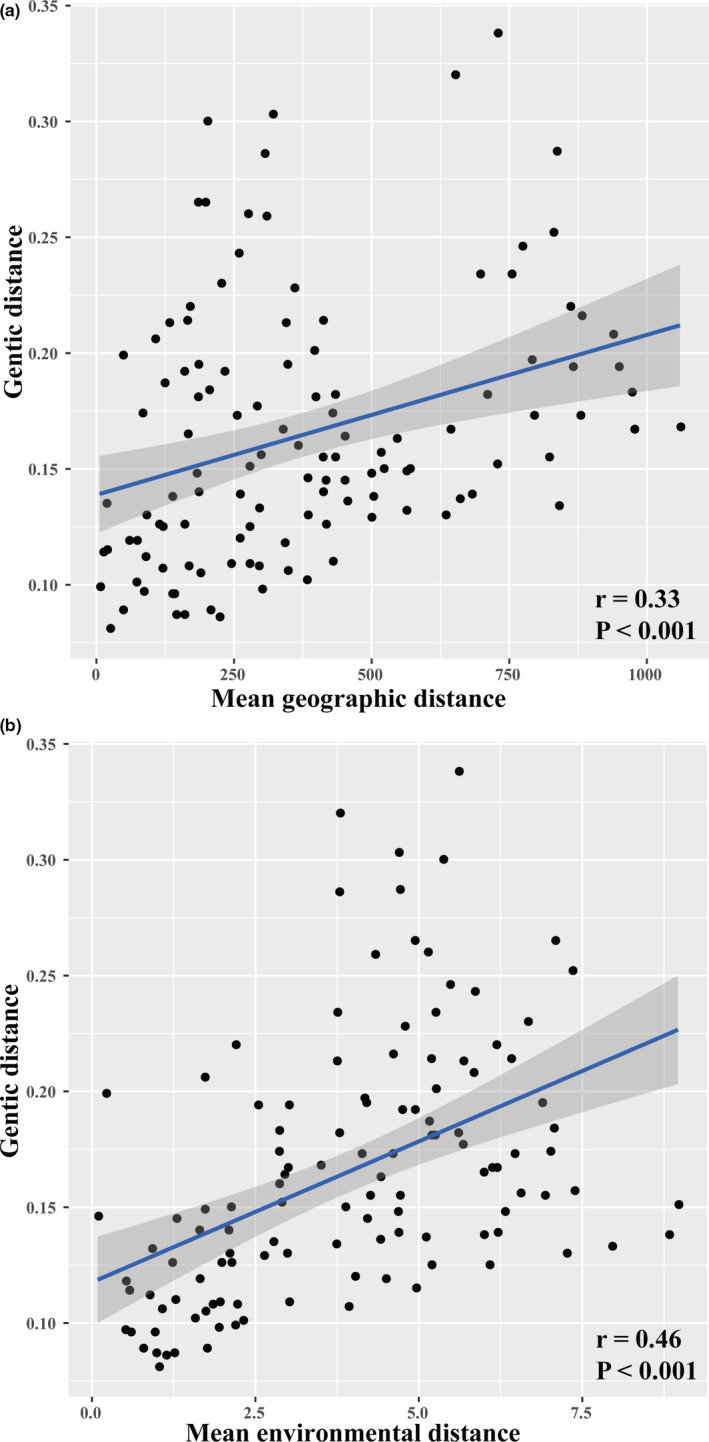
Correlations between geography, environment, and genetic data. The correlation of mean pairwise geographic distance versus mean pairwise *F*_ST_ (a), and correlation of mean pairwise environmental distance versus mean pairwise *F*_ST_ (b)

**FIGURE 5:**
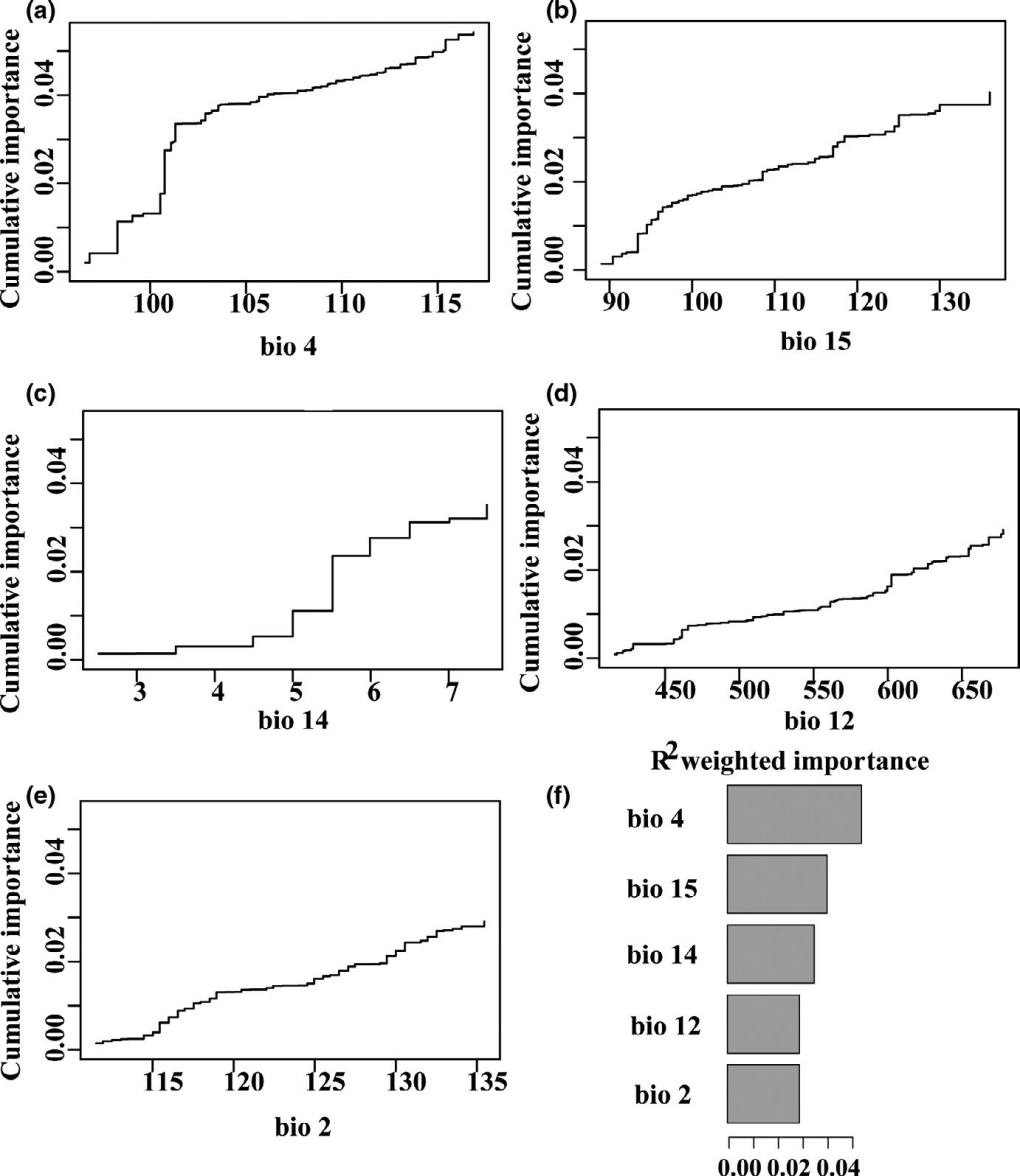
(a–e) Cumulative importance of genotype change along the five environmental gradients; (f) *R* ^2^‐weighted importance of environmental variables that explain genetic gradients

The IBD analyses indicated a significant correlation between geographic distance and genetic distance across populations (*r* = 0.33, *p* < 0.001) (Figure 4a). The first two principal components (Clim_PC1 and Clim_PC2) for all populations summarized 59.61% and 28.27% of the variation in the 19 climatic variables used in this study. A Mantel test among populations also determined significant correlation between genetic distance and environmental distance (*r* = 0.46, *p* < 0.001, Figure 4b). Our full RDA model indicated a significant role of geographic and environmental conditions in shaping the distribution of genotypes (*p* = 0.001; *R^2^* = 0.16). The first two RDA axes were significant and explained 12.20% of the constrained variance (Table S5). When single variable effects were conditioned by other variables effect in partial RDA analysis, our results revealed a significant effect of Clim_PC1 and Latitude on genomic variations (Table S6). Gradient forest analyses indicated that the most two important predictors were temperature seasonality and precipitation seasonality (Figure 5). Our results indicated a shift temperature seasonality (standard deviation × 100) was 101 and precipitation of driest month was 5.5 mm (Figure 5).

A total of eight and 21 *F*_ST_ outliers were identified in *M. dioica* using BayeScan and BayPass, respectively, with no overlapping outlier loci found between the two methods (Figures S4–S5). The BayPass analysis indicated a total of one and four loci significantly associated with Clim‐PC1 and Clim‐PC2, respectively (Figure S5). Based on the *H. annuus* genome, 22 loci associated with environment variables were successfully aligned to reference genome. A majority of the genes closest to the outlier loci were related to environmental stress response under drought tolerance, such as DREB and EAR motif protein 3, lipid transfer protein 3, and F‐box family protein (Table S6).

### 3.5 Joint effects of expansion dynamics and climatic factors on *Ne*

No association between the number of individuals sampled and *N_e_* was observed (*r* = −0.50, *p* = 0.07). We independently employed climate factors and population age for predicting *N_e_* in *M. dioica* populations. Our results showed no significant correlation between *N_e_* and population age (*r* = −0.24, *p* = 0.41, Figure S6). Although principal component analysis revealed that the first two cumulative contribution rates of variance for bioclimatic factors explained the majority of the variance, no significant correlation was found between PC1 of all climate factors and *N_e_* (*r* = −0.24, *p* = 0.42, Figure S6). In addition, there was no significant correlation between PC1 of energy/moisture and PC2 of energy/moisture to predict *N_e_* (Figure S7). The above results suggest that a single factor within either population history or climatic factors could not predict *N_e_* using a linear model. Thus, we further tested the multiple linear regression model of all the climate factors and population age. Due to the unevenness of the data, all variables were first normalized. Our stepwise regression showed the best fit linear model (*r^2^* = 0.51, *p* = 0.05, AIC = 35.46, Table 2) included population age, mean temperature of coldest quarter, and precipitation of wettest month.

**TABLE 2:** The optimal general linear models of species factors predicting effective population size *Ne*

Model	*Ne* ~ Bio11* + Bio13 + Population age
Multiple R‐squared	0.51
Akaike information criterion	35.46
*p*‐value	0.05

Note

“*” is corresponding to significant to *Ne*. Bio11 = mean temperature of coldest quarter; Bio13 = precipitation of wettest month.

## 4 DISCUSSION

### 4.1 Phylogeographic structure and demographic history

Our genomic data of *M. dioica* indicated a strong genetic population structure consisting of the northern and southern groups representing two distinct lineages geographically (Figure 1). Our best‐fitting ABC model identified the northern lineage as being ancestral and the southern group having been derived from it, which suggests that *M. dioica* originated north of the Taihang Mountains and then expanded south of the Taihang Mountains (Figure 3). The estimated divergence times for the southern and northern lineage occurring during the Pliocene to Pleistocene coincides with the intense uplift of the Taihang Mountains during the Late Pliocene to Pleistocene (Gong, 2010; Wu et al., 1999). With no detected migration events between the two groups, the division is inferred to be associated with restricted gene flow caused by long‐term isolation of geographic barrier. Visible mountains as barriers driving population subdivision and divergence have been reported in the Qinghai–Xizang Tibet Plateau and the Hengduan Mountains (Gao et al., 2003; Wu & Wu, 1996). The uplift of the Taihang Mountains during the Pliocene to Pleistocene, along with heterogeneous terrain and environmental change, undoubtedly promoted population divergence of plants in northern China flora, which has been indicated in previous results (Chai et al., 2020; Hou et al., 2014; Zhao et al., 2011). As phylogeographic information is useful in characterizing potential phylogeoregions, current genetic pattern of *M. dioica* and phylogeographical result in *Opisthopappus* suggests Taihang Mountains may be a potential phytogeographical boundary in Northern China (Chai et al., 2020). It is worth mentioning that we should be careful to estimate expansion and divergence time and population size changes from ABC model, as our data could not provide reliable distributions parameters and only provide a reference. The expansion–contraction model and in situ survival model are two alternative biogeographical models that have been applied to explain plant dynamics of the Quaternary period (Ni et al., 2010). When focusing on temperate forests in China, many studies have suggested the in situ survival model or the existence of multiple refugia in the northern regions during the Quaternary, such as in *Ostryopsis davidiana* Decne. (Tian et al., 2009) and *Juglans mandshurica* Maxim. (Bai et al., 2010). Our species distribution modeling revealed that no obvious southward contractions were found for *M. dioica* during the LGM, supporting the hypothesis of stable distribution by in situ survival. Analyses of the demographic history of the northern and southern group populations showed that both have experienced ancient expansions during the Pleistocene followed by bottlenecks during the recent warmer period (Figure 3). As affected by glaciation during the Pleistocene, temperature of the northern regions in China was significantly lower than those during the Pliocene and also became drier (Shi et al., 1999). This altered climate condition could be conducive to *M. dioica*, a cold and dry adapted species. Consequently, both geological events and climate oscillations contributed to phylogeographic pattern of *M. dioica*.

### 4.2 Genomic variations and climate‐driven adaptation

Our results based on IBE analyses indicated a significant effect of climatic factors on genetic differentiation of *M. dioica*, highlighting the contributions of environmental variables to the landscape genomics. Meanwhile, our RDA results indicated that the environment explained more genomic variations than geography, which is consistent with previous results (Orsini et al., 2013; Sexton et al., 2014). This also suggests that adaptation to the environment plays a key role in shaping plant divergence in the flora of northern China, and we used a landscape genomics approach to detect candidate genes for local adaption. A total of 22 SNPs were annotated to associate with climatic variables and a high proportion of located genes (over 50%) are involved in abiotic stress response of drought. Similar results have been presented in previous physiological studies in which *M. dioica* showed superior drought resistance of the root system (Dai et al., 2014; Xu et al., 2007). Among the outlier loci, we found genes involved in regulation of reduced lateral root formation. Plants decrease the metabolic cost of soil exploration, improving water acquisition and plant growth, which has been demonstrated to improve drought tolerance in *Zea mays* L. (Poaceae) and *Oryza glaberrima* Steud. (Poaceae) (Inukai et al., 2005; Lynch, 2013). Furthermore, we also detected loci in the super family of F‐box proteins and DREB, for which many studies have tested significant associations with drought in varied plant species (Ren et al., 2019; Zhang et al., 2008; Zhou et al., 2014). Consequently, current results based on *M. dioica* verified that drought stress is important and a dominant driver for local adaptation of plants in the flora of northern China, and *M. dioica* has undergone adaptive changes to better cope with an arid environment. Meanwhile, temperature seasonality and precipitation seasonality served as the most two crucial factors for the genomic variation of *M. dioica* among all the temperature and precipitation factors tested, and genetic composition shifts occurred when temperature seasonality was 101 and precipitation of driest month was 5.5 mm, which likely restricted the distribution margin of *M. dioica*.

### 4.3 Factors shaping limited effective population size and implications for conservation

To find evidence of the effects of both population history and climatic environment on populations evolution, we established relationships between effective population size across 16 populations and investigated factors being important for shaping evolutionary outcomes. Our results failed to indicate a significant positive correlation between a single factor (i.e., population age and expansion or climatic factors) and *N_e_*, which suggests that population history or climatic factors alone cannot predict the evolution of populations (Figure 5). Nevertheless, our multiple regression analysis showed that historical and environmental factors have complementary roles in explaining the evolutionary patterns of *M. dioica* (Table 2). First, we recognized population age of *M. dioica* to be one of the direct contributing factors for estimating population change (Braasch et al., 2019; Excoffier & Ray, 2008). Meanwhile, mean temperature of coldest quarter and precipitation of wettest month were shown to be important factors for population change in *M. dioica* inferring tolerance to coldness and drought allowing the species distribution in northern China (Xie & Zhang, 2012). Our current genome‐based linear model only offers a qualitative reference for predicting the population change of *M. dioica* and this specialized species evolved due to both population evolutionary history and climatic variables. As our genome‐based result offers a qualitative guideline for predicting population change, it is easy to screen factors directly related to population evolution. This will be highly effective to providing guidance for endemic plant of narrow area.

Although *M. dioica* are distributed throughout northern China, it is particularly troubling that many specimen locations from previous records cannot be found, and only a very few individuals are left in some places during our filed investigations. In this case, *M. dioica* may be currently under threat and it is necessary to take measures in advance to prevent further disappearance. *Myripnois dioica* is usually distributed in disturbed, open habitats, occurring on slopes of limestone, and even along the roadside. Human activities have caused species diversity loss by habitat destruction, suggesting that *M. dioica* habitats are under constant threat with some populations already having been extirpated (Chittibabu & Parthasarathy, 2000; Fang et al., 2018). Meanwhile, the clear result in the current study is the distinct isolation between southern and northern lineages which are almost certainly not interconnected by gene flow. Given the distinct two lineages among *M. dioica* populations which have adapted to the local ecology, overpromoting gene flow between populations may lead to outbreeding depression and decreased fitness. A recent study suggested that intraspecific gene flow between edge populations potentially facilitates population conservation (Hannah et al., 2014). In addition to placing attention to populations around the genetic hot spots, we suggest a background survey of wild populations of *M. dioica* including the total number of populations, genetic differentiation between populations, intermediate/edge populations should be implemented across its entire range. Combined with our predicted evolution factors, these will help to adequately address accurate conservation strategies of *M. dioica* with significant reference to other species responding to climatic change and conservation management.

## AUTHOR CONTRIBUTIONS

**Nan Lin:** Data curation (lead); Formal analysis (lead); Investigation (lead); Methodology (lead); Software (lead); Writing‐original draft (lead). **Jacob B. Landis:** Formal analysis (supporting); Methodology (supporting); Software (supporting); Writing‐review & editing (supporting). **Yanxia Sun:** Methodology (supporting); Software (supporting). **Xianhan Huang:** Methodology (supporting); Software (supporting). **Xu Zhang:** Investigation (supporting); Resources (supporting). **Qun Liu:** Investigation (supporting); Resources (supporting). **Huajie Zhang:** Investigation (supporting); Resources (supporting). **Hang Sun:** Conceptualization (equal); Funding acquisition (equal); Supervision (equal); Writing‐review & editing (equal). **Hengchang Wang:** Conceptualization (equal); Supervision (equal); Writing‐review & editing (equal). **Tao Deng:** Conceptualization (equal); Funding acquisition (equal); Supervision (equal); Writing‐review & editing (equal).

## Supporting information

Supplementary Material

## Data Availability

The raw data were deposited in the NCBI Short Read Archive (SRA) (Bioproject No. PRJNA607823).

## References

[ece37628-bib-0001] Alexander, D. H. , Novembre, J. , & Lange, K. (2009). Fast model‐based estimation of ancestry in unrelated individuals. Genome Research, 19, 1655–1664. 10.1101/gr.094052.109 19648217 PMC2752134

[ece37628-bib-0002] Badouin, H. , Gouzy, J. , Grassa, C. J. , Murat, F. , Staton, S. E. , Cottret, L. , Lelandais‐Brière, C. , Owens, G. L. , Carrère, S. , Mayjonade, B. , Legrand, L. , Gill, N. , Kane, N. C. , Bowers, J. E. , Hubner, S. , Bellec, A. , Bérard, A. , Bergès, H. , Blanchet, N. , … Langlade, N. B. (2017). The sunflower genome provides insights into oil metabolism, flowering and Asterid evolution. Nature, 546, 148–152. 10.1038/nature22380 28538728

[ece37628-bib-0003] Bai, W. N. , Liao, W. J. , & Zhang, D. Y. (2010). Nuclear and chloroplast DNA phylogeography reveal two refuge areas with asymmetrical gene flow in a temperate walnut tree from East Asia. New Phytologist, 188, 892–901. 10.1111/j.1469-8137.2010.03407.x 20723077

[ece37628-bib-0004] Bai, W. N. , Wang, W. T. , & Zhang, D. Y. (2016). Phylogeographic breaks within Asian butternuts indicate the existence of a phytogeographic divide in East Asia. New Phytologist, 209, 1757–1772. 10.1111/nph.13711 26499508

[ece37628-bib-0005] Basu, S. , Ramegowda, V. , Kumar, A. , & Pereira, A. (2016). Plant adaptation to drought stress. F1000Research, 5, 1554. 10.12688/f1000research.7678.1 PMC4937719 27441087

[ece37628-bib-0006] Braasch, J. , Barker, B. S. , & Dlugosch, K. M. (2019). Expansion history and environmental suitability shape effective population size in a plant invasion. Molecular Ecology, 28, 2546–2558. 10.1111/mec.15104 30993767 PMC6584048

[ece37628-bib-0007] Cao, X. Y. , Herzschuh, U. , Ni, J. , Zhao, Y. , & Boehmer, T. (2015). Spatial and temporal distributions of major tree taxa in eastern continental Asia during the last 22,000 years. Holocene, 25, 79–91. 10.1177/0959683614556385

[ece37628-bib-0008] Catchen, J. , Hohenlohe, P. A. , Bassham, S. , Amores, A. , & Cresko, W. A. (2013). Stacks: An analysis tool set for population genomics. Molecular Ecology, 22, 3124–3140. 10.1111/mec.12354 23701397 PMC3936987

[ece37628-bib-0009] Chai, M. , Ye, H. , Wang, Z. , Zhou, Y. C. , Wu, J. H. , Gao, Y. , Han, W. , Zang, E. , Zhang, H. , Ru, W. M. , Sun, G. L. , & Wang, Y. L. (2020). Genetic divergence and relationship among *Opisthopappus* species identified by development of EST‐SSR Markers. Frontiers in Genetics, 11, 177. 10.3389/fgene.2020 32194635 PMC7065708

[ece37628-bib-0010] Chao, F. , Wang, J. , Wu, L. Q. , Kong, H. H. , Yang, L. H. , Feng, C. , Wang, K. , Rausher, M. , & Kang, M. (2020). The genome of a cave plant, *Primulina huaijiensis*, provides insights into adaptation to limestone karst habitats. New Phytologist, 227, 1249–1263. 10.1111/nph.16588 32274804

[ece37628-bib-0011] Chen, K. M. , Abbott, R. J. , Milne, R. I. , Tian, X. M. , & Liu, J. Q. (2008). Phylogeography of *Pinus tabulaeformis* Carr. (Pinaceae), a dominant species of coniferous forest in northern China. Molecular Ecology, 17, 4276–4288. 10.1111/j.1365-294X.2008.03911.x 19378405

[ece37628-bib-0012] Chen, Y. S. , Deng, T. , Zhou, Z. , & Sun, H. (2018). Is the East Asian flora ancient or not? National Science Review, 5, 920–932. 10.1093/nsr/nwx156

[ece37628-bib-0013] Chittibabu, C. , & Parthasarathy, N. (2000). Attenuated tree species diversity in human‐impacted tropical evergreen forest sites at Kolli hills, Eastern Ghats, India. Biodiversity and Conservation, 9, 1493–1519. 10.1023/A:1008971015545

[ece37628-bib-0014] Cornuet, J. M. , Pudlo, P. , Veyssier, J. , Dehne‐Garcia, A. , Gautier, M. , Leblois, R. , Marin, J. M. , & Estoup, A. (2014). DIYABC v2.0: A software to make approximate Bayesian computation inferences about population history using single nucleotide polymorphism. DNA sequence and microsatellite data. Bioinformatics, 30, 1187–1189. 10.5061/dryad.h70rx 24389659

[ece37628-bib-0015] Dai, L. F. , Zhang, Z. X. , & Shen, Y. B. (2014). A study on shade tolerance of four shrub species. Journal of West China Forestry Science, 36, 41–48.

[ece37628-bib-0016] Danecek, P. , Auton, A. , Abecasis, G. , Albers, C. A. , Banks, E. , DePristo, M. A. , Handsaker, R. E. , Lunter, G. , Marth, G. T. , Sherry, S. T. , McVean, G. , & Durbin, R. (2011). The variant call format and VCFtools. Bioinformatics, 27, 2156–2158. 10.1093/bioinformatics/btr330 21653522 PMC3137218

[ece37628-bib-0017] De Kort, H. , Vandepitte, K. , Bruun, H. H. , Closset‐Kopp, D. , Honnay, O. , & Mergeay, J. (2014). Landscape genomics and a common garden trial reveal adaptive differentiation to temperature across Europe in the tree species *Alnus glutinosa* . Molecular Ecology, 23, 4709–4721. 10.3864/j.issn.0578-1752.2013.14.021 24860941

[ece37628-bib-0018] Do, C. , Waples, R. S. , Peel, D. , Macbeth, G. M. , Tillett, B. J. , & Ovenden, J. R. (2014). NEESTIMATOR v2: Re‐implementation of software for the estimation of contemporary effective population size (*N*e)) from genetic data. Molecular Ecology Resources, 14, 209–214. 10.1111/1755-0998.12157 23992227

[ece37628-bib-0019] Doyle, J. J. , Doyle, J. L. , Ballenger, J. A. , & Palmer, J. D. (1996). The distribution and phylogenetic significance of a 50‐kb chloroplast DNA inversion in the flowering plant family Leguminosae. Molecular Phylogenetics and Evolution, 5, 429–438. 10.1006/mpev.1996.0038 8728401

[ece37628-bib-0020] Drummond, A. J. , & Rambaut, A. (2007). BEAST: Bayesian evolutionary analysis by sampling trees. BMC Evolutionary Biology, 7, 214. 10.1186/1471-2148-7-214 17996036 PMC2247476

[ece37628-bib-0021] Ellis, N. , Smith, S. J. , & Pitcher, C. R. (2012). Gradient forests: Calculating importance gradients on physical predictors. Ecology, 93, 156–168. 10.1890/11-0252.1 22486096

[ece37628-bib-0022] Excoffier, L. , Laval, G. , & Schneider, L. (2005). Arlequin (version 3.0): An integrated software package for population genetics data analysis. Evolution Bioinformatics, 1, 47–50. 10.1177/117693430500100003 PMC2658868 19325852

[ece37628-bib-0023] Excoffier, L. , & Ray, N. (2008). Surfing during population expansions promotes genetic revolutions and structuration. Trends in Ecology and Evolution, 23, 347–351. 10.1111/mec.14988 18502536

[ece37628-bib-0024] Fan, L. Q. , Zheng, H. L. , Milne, R. I. , Zhang, L. , & Mao, K. S. (2018). Strong population bottleneck and repeated demographic expansions of *Populus adenopoda* (Salicaceae) in subtropical China. Annals of Botany, 121, 665–679. 10.1093/aob/mcx198 29324975 PMC5853028

[ece37628-bib-0025] Fang, J. Y. , Yu, G. , Liu, L. , Hu, S. , & Chapin, F. S. (2018). Climate change, human impact, and carbon sequestration in China. Proceedings of the National Academy of Sciences of the United States of America, 115, 4015–4020. 10.1073/pnas.1700304115 29666313 PMC5910806

[ece37628-bib-0026] Foll, M. , & Gaggiotti, O. (2008). A genome‐scan method to identify selected loci appropriate for both dominant and codominant markers: A Bayesian perspective. Genetics, 180, 977–993. 10.1534/genetics.108.092221 18780740 PMC2567396

[ece37628-bib-0027] Funk, V. A. , Sancho, G. , Roque, N. , Kelloff, C. L. , Ventosa‐Rodriguez, I. , Diazgranados, M. , Bonifacino, J. M. , & Chan, R. (2014). A phylogeny of the Gochnatieae: Understanding a critically placed tribe in the Compositae. Taxon, 63, 859–882. 10.1016/j.ympev.2018.04.033

[ece37628-bib-0028] Gao, Q. , Li, X. B. , & Yang, X. S. (2003). Responses of vegetation and primary production in north–south transect of eastern China to global change under land use constraint. Acta Botanica Sinica, 45, 1274–1284.

[ece37628-bib-0029] Gao, T. G. , & Nicholas, D. J. (2011). *Myripnois* Bunge, Enum. Pl. China Bor. 38. 1833. Flora of China, 21, 31–32.

[ece37628-bib-0030] Gautier, M. (2015). Genome‐wide scan for adaptive divergence and association with population‐specific covariates. Genetics, 201, 1555–1579. 10.1534/genetics.115.181453 26482796 PMC4676524

[ece37628-bib-0031] Gong, M. (2010). Uplifting process of Southern Taihang Mountain in Cenozoic. PhD Thesis, Chinese Academy of Geological Science, Beijing, China.

[ece37628-bib-0032] Guo, Z. T. , Sun, B. , Zhang, Z. S. , Peng, S. Z. , Xiao, G. Q. , Ge, J. Y. , Hao, Q. Z. , Qiao, Y. S. , Liang, M. Y. , Liu, J. F. , Yin, Q. Z. , & Wei, J. J. (2008). A major reorganization of Asian climate by the early Miocene. Climate of the Past, 4, 153–174. 10.5194/cp-4-153-2008

[ece37628-bib-0033] Hannah, L. , Flint, L. , Syphard, A. D. , Moritz, M. A. , Buckley, L. B. , & McCullough, I. M. (2014). Fine‐grain modeling of species' response to climate change: Holdouts, stepping‐stones, and microrefugia. Trends in Ecology and Evolution, 29, 390–397. 10.1016/j.tree.2014.04.006 24875589

[ece37628-bib-0034] Hao, Q. , de Lafontaine, G. , Guo, D. S. , Gu, H. Y. , Hu, F. S. , Han, Y. , Song, Z. , & Liu, H. Y. (2018). The critical role of local refugia in postglacial colonization of Chinese pine: Joint inferences from DNA analyses, pollen records, and species distribution modeling. Ecography, 41, 592–606. 10.1111/mec.13477

[ece37628-bib-0035] Haselhorst, M. S. H. , Parchman, T. L. , & Buerkle, C. A. (2019). Genetic evidence for species cohesion, substructure and hybrids in spruce. Molecular Ecology, 28, 2029–2045. 10.1111/mec.15056 30801841

[ece37628-bib-0036] Hasumi, H. , & Emori, S. (2004). K‐1 coupled GCM (MIROC) description. Center for Climate System Research, University of Tokyo.

[ece37628-bib-0037] Hijmans, R. J. , Cameron, S. E. , Parra, J. L. , Jones, P. G. , & Jarvis, A. (2005). Very high resolution interpolated climate surfaces for global land areas. International Journal of Climatology, 25, 1965–1978. 10.1002/joc.1276

[ece37628-bib-0038] Hou, H. , Ye, H. , Wang, Z. , Wu, J. , Gao, Y. , Han, W. , Na, D. , Sun, G. , & Wang, Y. (2020). Demographic history and genetic differentiation of an endemic and endangered *Ulmus lamellosa* (Ulmus). BMC Plant Biology, 20, 526. 10.1186/s12870-020-02723-7 33203402 PMC7672979

[ece37628-bib-0039] Hou, Z. , Li, J. , & Li, S. (2014). Diversification of low dispersal crustaceans through mountain uplift: A case study of Gammarus (Amphipoda: Gammaridae) with descriptions of four novel species. Zoological Journal of the Linnean Society, 170, 591–633. 10.1111/zoj.12119

[ece37628-bib-0040] Inukai, Y. , Sakamoto, T. , Ueguchi‐Tanaka, M. , Shibata, Y. , Gomi, K. , Umemura, I. , Hasegawa, Y. , Ashikari, M. , Kitano, H. , & Matsuoka, M. (2005). *Crown rootless 1*, which is essential for crown root formation in rice, is a target of an AUXIN RESPONSE FACTOR in auxin signaling. The Plant Cell, 175, 1387–1396. 10.1105/tpc.105.030981 PMC1091762 15829602

[ece37628-bib-0041] Jarzyna, M. , & Jetz, W. (2016). Detecting the multiple facets of biodiversity. Trends in Ecology and Evolution, 31, 527–538. 10.1016/j.tree.2016.04.002 27168115

[ece37628-bib-0042] Jones, M. R. , Forester, B. R. , Teufel, A. I. , Adams, R. V. , Anstett, D. N. , Goodrich, B. A. , Landguth, E. L. , Joost, S. , & Manel, S. (2013). Integrating landscape genomics and spatially explicit approaches to detect loci under selection in clinal populations. Evolution: International Journal of Organic Evolution, 67, 3455–3468. 10.1111/evo.12237 24299400

[ece37628-bib-0043] Joost, S. , Bonin, A. , Bruford, M. W. , Després, L. , Conord, C. , Erhardt, G. , & Taberlet, P. A. (2007). Spatial analysis method (SAM) to detect candidate loci for selection: Towards a landscape genomics approach to adaptation. Molecular Ecology, 16, 3955–3969. 10.1111/j.1365-294X.2007.03442.x 17850556

[ece37628-bib-0045] Li, J. J. , Fang, X. M. , Pan, B. T. , Zhao, Z. J. , & Song, Y. G. (2001). Late Cenozoic intensive uplift of Qinghai‐Xizang Plateau and its impacts on environments in surrounding area. Quaternary Sciences, 21, 381–391. 10.3321/j.issn:1001-7410.2001.05.001

[ece37628-bib-0046] Li, Y. , Zhang, X. X. , Mao, R. L. , Yang, J. , Miao, C. Y. , Li, Z. , & Qiu, X. Y. (2017). Ten years of landscape genomics: Challenges and opportunities. Frontiers in Plant Science, 8, 2136. 10.3389/fpls.2017.02136 29312391 PMC5733015

[ece37628-bib-0047] Lischer, H. E. L. , & Excoffier, L. (2012). PGDSpider: An automated data conversion tool for connecting population genetics and genomics programs. Bioinformatics, 28, 298–299. 10.1093/bioinformatics/btr642 22110245

[ece37628-bib-0048] Lu, L.‐M. , Mao, L.‐F. , Yang, T. , Ye, J.‐F. , Liu, B. , Li, H.‐L. , Sun, M. , Miller, J. T. , Mathews, S. , Hu, H.‐H. , Niu, Y.‐T. , Peng, D.‐X. , Chen, Y.‐H. , Smith, S. A. , Chen, M. , Xiang, K.‐L. , Le, C.‐T. , Dang, V.‐C. , Lu, A.‐M. , … Chen, Z.‐D. (2018). Evolutionary history of the angiosperm flora of China. Nature, 554, 234–238. 10.1038/nature25485 29420476

[ece37628-bib-0049] Lynch, J. P. (2013). Steep, cheap and deep: An ideotype to optimize water and N acquisition by maize root systems. Annals of Botany, 112, 347–357. 10.1093/aob/mcs293 23328767 PMC3698384

[ece37628-bib-0050] Manchester, S. R. , Chen, Z. D. , Lu, A. M. , & Uemura, K. (2009). Eastern Asian endemic seed plant genera and their paleogeographic history throughout the Northern Hemisphere. Journal of Systematics and Evolution, 47, 1–42. 10.1007/s00122-008-0717-3

[ece37628-bib-0051] Manel, S. , Gugerli, F. , Thuiller, W. , Alvarez, N. , Legendre, P. , Holderegger, R. , Gielly, L. , & Taberlet, P. (2012). Broad‐scale adaptive genetic variation in alpine plants is driven by temperature and precipitation. Molecular Ecology, 21, 3729–3738. 10.1111/j.1365-294X.2012.05656.x 22680783 PMC4003392

[ece37628-bib-0052] Médail, F. , & Baumel, A. (2018). Using phylogeography to define conservation priorities: The case of narrow endemic plants in the Mediterranean Basin hotspot. Biological Conservation, 224, 258–266. 10.1016/j.biocon.2018.05.028

[ece37628-bib-0053] Micheletti, S. J. , & Storfer, A. (2015). A test of the central–marginal hypothesis using population genetics and ecological niche modelling in an endemic salamander (*Ambystoma barbouri*). Molecular Ecology, 24, 967–979. 10.1186/s12864-018-5346-x 25604892

[ece37628-bib-0054] Moritz, C. C. , & Potter, S. (2013). The importance of an evolutionary perspective in conservation policy planning. Molecular Ecology, 22, 5969–5971. 10.1111/mec.12565 24138133

[ece37628-bib-0055] Ni, J. , Yu, G. , Harrison, S. P. , & Prentice, I. C. (2010). Palaeovegetation in China during the late Quaternary: Biome reconstructions based on a global scheme of plant functional types. Palaeogeography Palaeoclimatology Palaeoecology, 289, 44–61. 10.1016/j.palaeo.2010.02.008

[ece37628-bib-0056] Oksanen, J. , Blanchet, F. G. , Friendly, M. , Kindt, R. , Legendre, P. , McGlinn, D. , Minchin, P. R. , O’Hara, R. B. , Simpson, G. L. , Solymos, P. , Stevens, M. H. H. , Szoecs, E. , & Wagner, H. (2020). vegan community ecology package version 2.5‐7 November 2020.

[ece37628-bib-0057] Orsini, L. , Vanoverbeke, J. , Swillen, I. , Mergeay, J. , & De Meester, L. (2013). Drivers of population genetic differentiation in the wild: Isolation by dispersal limitation, isolation by adaptation and isolation by colonization. Molecular Ecology, 22, 5983–5999. 10.1038/s41598-018-31938-w 24128305

[ece37628-bib-0058] Pauls, S. U. , Nowak, C. , Balint, M. , & Pfenninger, M. (2013). The impact of global climate change on genetic diversity within populations and species. Molecular Ecology, 22, 925–946. 10.1111/mec.12152 23279006

[ece37628-bib-0059] Peakall, R. O. D. , & Smouse, P. E. (2006). GENALEX 6: Genetic analysis in Excel. Population genetic software for teaching and research. Molecular Ecology Notes, 6, 288–295. 10.1111/j.1471-8286.2005.01155.x PMC3463245 22820204

[ece37628-bib-0060] Peres‐Neto, P. R. , Legendre, P. , Dray, S. , & Borcard, D. (2006). Variation partitioning of species data matrices: Estimation and comparison of fractions. Ecology, 87, 2614–2625. 10.1890/0012-9658(2006)87 17089669

[ece37628-bib-0061] Phillips, S. J. , Anderson, R. P. , & Schapire, R. E. (2006). Maximum entropy modeling of species geographic distributions. Ecological Modelling, 190, 231–259. 10.1016/j.ecolmodel.2005.03.026

[ece37628-bib-0062] Pickrell, J. K. , & Pritchard, J. K. (2012). Inference of population splits and mixtures from genome‐wide allele frequency data. PLoS Genetics, 8, e1002967. 10.1371/journal.pgen.1002967 23166502 PMC3499260

[ece37628-bib-0063] Pluess, A. R. , Frank, A. , Heiri, C. , Lalague, H. , Vendramin, G. G. , & Oddou‐Muratorio, S. (2016). Genome–environment association study suggests local adaptation to climate at the regional scale in *Fagus sylvatica* . New Phytologist, 210, 589–601. 10.1111/nph.13809 26777878

[ece37628-bib-0064] Polechová, J. , & Storch, D. (2008). Ecological niche. In S. K. Jørgensen , & B. D. Fath (Eds.), Encyclopedia of ecology (Vol. 2, pp. 1088–1097). Elsevier.

[ece37628-bib-0065] Pritchard, J. K. , Stephens, M. , & Donnelly, P. (2000). Inference of population structure using multilocus genotype data. Genetics, 155, 945–959. 10835412 10.1093/genetics/155.2.945 PMC1461096

[ece37628-bib-0066] R Core Team (2013). R: A language and environment for statistical computing. R Foundation for Statistical Computing.

[ece37628-bib-0067] Ren, M. , Wang, Z. , Xue, M. , Wang, X. , Zhang, F. , Zhang, Y. U. , Zhang, W. , & Wang, M. (2019). Constitutive expression of an A‐5 subgroup member in the DREB transcription factor subfamily from *Ammopiptanthus mongolicus* enhanced abiotic stress tolerance and anthocyanin accumulation in transgenic *Arabidopsis* . PLoS One, 14, e0224296. 10.1371/journal.pone.0224296 31644601 PMC6808444

[ece37628-bib-0068] Ronquist, F. , & Huelsenbeck, J. P. (2003). MrBayes 3: Bayesian phylogenetic inference under mixed models. Bioinformatics, 19, 1572–1574. 10.1093/bioinformatics/btg180 12912839

[ece37628-bib-0069] Sexton, J. P. , Hangartner, S. B. , & Hoffmann, A. A. (2014). Genetic isolation by environment or distance: Which pattern of gene flow is most common? Evolution, 68, 1–15. 10.1111/evo.12258 24111567

[ece37628-bib-0070] Shi, Y. F. , Li, J. Y. , Li, B. Y. , Yao, T. D. , Wang, S. M. , Li, S. J. , Tsui, Z. J. , Wang, F. B. , Pan, B. T. , Fang, X. M. , & Zhang, Q. S. (1999). Uplift of the Qinghai‐Xizang (Tibetan) plateau and east Asia environmental change during late Cenozoic. Acta Geograph, 54, 10–21. 10.1007/s11295-016-0968-0

[ece37628-bib-0071] Stamatakis, A. (2014). RAxML version 8: A tool for phylogenetic analysis and post‐analysis of large phylogenies. Bioinformatics, 30, 1312–1313. 10.1111/mec.14994 24451623 PMC3998144

[ece37628-bib-0072] Tang, C. Q. , Matsui, T. , Ohashi, H. , Dong, Y. F. , Momohara, A. , Herrando‐Moraira, S. , Qian, S. , Yang, Y. , Ohsawa, M. , Luu, H. T. , Grote, P. J. , Krestov, P. V. , LePage, B. , Werger, M. , Robertson, K. , Hobohm, C. , Wang, C. Y. , Peng, M. C. , Chen, X. I. , … López‐Pujol, J. (2018). Identifying long‐term stable refugia for relict plant species in East Asia. Nature Communications, 9, 4488. 10.1038/s41467-018-06837-3 PMC6203703 30367062

[ece37628-bib-0073] Tian, B. , Liu, R. , Wang, L. , Qiu, Q. , Chen, K. , & Liu, J. (2009). Phylogeographic analyses suggest that a deciduous species (*Ostryopsis davidiana* Decne., Betulaceae) survived in northern China during the Last Glacial Maximum. Journal of Biogeography, 36, 2148–2155. 10.1111/j.1365-2699.2009.02157.x

[ece37628-bib-0074] Wang, H. C. (1999). Evolution and origin of northern China flora. Journal of Geographical Sciences, 54, 213–223.

[ece37628-bib-0075] Wang, Q. , Liu, J. , Allen, G. A. , Ma, Y. , Yue, W. , Marr, K. L. , & Abbott, R. J. (2016). Arctic plant origins and early formation of circumarctic distributions: A case study of the mountain sorrel, *Oxyria digyna* . New Phytologist, 209, 343–353. 10.1111/nph.13568 26197783

[ece37628-bib-0076] Wang, Z. , Zeng, Y. , Zhang, Z. , Sheng, S. , Tian, J. , Wu, R. , & Pang, X. (2017). Phylogeography study of the *Siberian Apricot* (*Prunus sibirica* L.) in Northern China assessed by chloroplast microsatellite and DNA Makers. Frontiers. Plant Science, 8, 1989. 10.3389/fpls.2017.01989 PMC5702509 29209348

[ece37628-bib-0077] Warschefsky, E. J. , & von Wettberg, E. J. B. (2019). Population genomic analysis of mango (*Mangifera indica*) suggests a complex history of domestication. New Phytologist, 222, 2023–2037. 10.1111/nph.15731 30730057

[ece37628-bib-0078] Wu, C. , Zhang, X. , & Ma, Y. (1999). The Taihang and Yan mountains rose mainly in Quaternary. North China Earthquake Sciences, 17, 1–7. 10.1007/s11103-011-9753-5

[ece37628-bib-0079] Wu, Z. Y. , Sun, H. , Zhou, Z. K. , Peng, H. , & Li, D. Z. (2005). Origin and differentiation of endemism in the flora of China. Acta Botanica Yunnanica, 27, 577–604. 10.1007/s11515-007-0020-8

[ece37628-bib-0080] Wu, Z. Y. , & Wu, S. G. (1996). A proposal for a new floristic kingdom (realm): The East Asiatic Kingdom, its delineation and characteristics. In A. L. Zhang , & S. G. Wu (Eds.), Floristic characteristics and diversity of East Asian plants, proceedings of the first international symposium on floristic characteristics and diversity of East Asian plants (pp. 3–42). China Higher Education Press.

[ece37628-bib-0081] Xie, Q. Z. , & Zhang, X. H. (2012). *Myripnois dioica*: A beautiful woody dwarf plant mater. Journal of Chinese Urban Forestry, 10, 56–57. 10.3969/j.issn.1672-4925.2012.05.021

[ece37628-bib-0082] Xu, X. Y. , Zhang, F. J. , Wang, Z. H. , Yin, W. L. , & Wang, H. F. (2007). Relationship between drought resistance and leaf water balance ability of six shrubs in Yanshan area. Acta Botanica Boreali‐Occidentalia Sinica, 27, 2080–2088.

[ece37628-bib-0083] Ye, H. , Wu, J. , Hou, H. , Wang, Z. , & Wang, Y. (2018). Population differentiation of endangered *Opisthopappus* Shih in China. Abstracts of papers of the 85th annual meeting of Chinese botanical society.

[ece37628-bib-0084] Ye, J. W. , Zhang, Z. K. , Wang, H. F. , Bao, L. , & Ge, J. P. (2019). Phylogeography of *Schisandra chinensis* (Magnoliaceae) reveal multiple refugia with ample gene flow in Northeast China. Frontiers in Plant Science, 10, 199. 10.3389/fpls.2019.00199 30858859 PMC6397880

[ece37628-bib-0085] Yu, G. , Chen, X. , Ni, J. , Cheddadi, R. , Guiot, J. , Han, H. , & Zheng, Z. (2000). Palaeovegetation of China: A pollen data‐based synthesis for the mid‐Holocene and last glacial maximum. Journal of Biogeography, 27, 635–666. 10.1007/s00606-013-0859-x

[ece37628-bib-0086] Zhang, Y. E. , Xu, W. , Li, Z. , Deng, X. W. , Wu, W. , & Xue, Y. (2008). F‐box protein DOR functions as a novel inhibitory factor for abscisic acid‐induced stomatal closure under drought stress in *Arabidopsis* . Plant Physiology, 148, 2121–2133. 10.1104/pp.108.126912 18835996 PMC2593669

[ece37628-bib-0087] Zhao, Q. , Liu, H. X. , Luo, L. G. , & Ji, X. (2011). Comparative population genetics and phylogeography of two lacertid lizards (*Eremias argus* and E. brenchleyi) from China. Molecular Phylogenetics and Evolution, 58, 478–491. 10.1016/j.ympev.2010.12.017 21215808

[ece37628-bib-0088] Zhao, Y. J. , & Gong, X. (2015). Genetic divergence and phylogeographic history of two closely related species (*Leucomeris decora* and *Nouelia insignis*) across the ‘Tanaka Line’ in Southwest China. BMC Evolutionary Biology, 15, 134. 10.1186/s12862-015-0374-5 26153437 PMC4495643

[ece37628-bib-0089] Zhou, S. M. , Sun, X. D. , Yin, S. H. , Kong, X. Z. , Zhou, S. , Xu, Y. , Luo, Y. , & Wang, W. (2014). The role of the F‐box gene TaFBA1 from wheat (*Triticum aestivum* L.) in drought tolerance. Plant Physiology and Biochemistry, 84, 213–223. 10.13560/j.cnki.biotech.bull.1985.2016.01.013 25299612

