# Supplementary material for: Demographic history and local adaptation of Myripnois dioica (Asteraceae) provide insight on plant evolution in northern China flora
Source: Ecol Evol. 2021 May 17;11(12):8000–13. doi: 10.1002/ece3.7628 (PMC8216978; doi:10.1002/ece3.7628)
Supplement: Supplementary file 1 — Supplementary Material [file ECE3-11-8000-s001.docx]

**Appendixes**

Table S1 Detailed GPS information and voucher specimen of each population.

| Population | Longitude (^。^E) | Latitude (^。^N) | Altitude (m) | Voucher specimen |
| --- | --- | --- | --- | --- |
| BA | 113.05 | 35.35 | 858 | HIB-NL180723 |
| FZ | 113.57 | 39.26 | 1100 | HIB-NL180731 |
| GSJ | 113.57 | 35.55 | 646 | HIB-NL180724 |
| HH | 120.04 | 41.17 | 671 | HIB-NL180728 |
| HX | 113.99 | 35.65 | 595 | HIB-NL180725 |
| HY | 113.71 | 39.66 | 1227 | HIB-NL180726 |
| JC | 111.66 | 37.68 | 1346 | HIB-NL180729 |
| JH | 121.06 | 41.18 | 204 | HIB-NL180727 |
| JL | 116.04 | 39.95 | 651 | HIB-NL180802 |
| JW | 112.44 | 37.97 | 1027 | HIB-NL180730 |
| MF | 116.03 | 40.07 | 875 | HIB-NL180807 |
| QHD | 119.47 | 40.20 | 252 | HIB-NL180804 |
| TL | 112.42 | 37.71 | 904 | HIB-NL180806 |
| XS | 116.20 | 40.00 | 196 | HIB-NL180803 |
| YJP | 115.39 | 40.07 | 1314 | HIB-NL180802 |
| YX | 114.97 | 39.94 | 1429 | HIB-NL180801 |

Table S2 Summary of genomic data collected for each population. N_SITES, *H*_O_, *H*_E_ and N_MISS corresponds to the number of SNPs, a measure of observed heterozygosity, expected heterozygosity and amount of missing data for each individual.

| Individuals | N_SITES | *H*_O_ | *H_E_* | N_MISS |
| --- | --- | --- | --- | --- |
| BA1 | 12542 | 12299 | 11187.5 | 10326 |
| BA2 | 9867 | 8699 | 7825.6 | 11673 |
| BA3 | 13800 | 11866 | 10927.8 | 7740 |
| BA4 | 13272 | 11473 | 10506.4 | 8268 |
| BA5 | 13762 | 11745 | 10895.4 | 7778 |
| FZ1 | 18577 | 14664 | 14717.9 | 2963 |
| FZ2 | 19242 | 15310 | 15242.6 | 2298 |
| FZ3 | 18863 | 14718 | 14940.3 | 2677 |
| FZ4 | 16140 | 13030 | 12798.2 | 5400 |
| FZ5 | 18885 | 15157 | 14956.7 | 2655 |
| FZ6 | 19000 | 15110 | 15057.7 | 2540 |
| FZ7 | 17840 | 14327 | 14142.7 | 3700 |
| GSJ1 | 17640 | 14755 | 13979 | 3900 |
| GSJ2 | 16604 | 13918 | 13152.7 | 4936 |
| GSJ3 | 17797 | 14905 | 14104.8 | 3743 |
| GSJ4 | 17507 | 14504 | 13876.8 | 4033 |
| GSJ5 | 16922 | 14080 | 13411.7 | 4618 |
| GSJ6 | 17306 | 14238 | 13710.8 | 4234 |
| HH1 | 18146 | 14313 | 14382.7 | 3394 |
| HH2 | 17823 | 14029 | 14131 | 3717 |
| HH3 | 18277 | 14409 | 14483.4 | 3263 |
| HX1 | 18397 | 15088 | 14558.9 | 3143 |
| HX2 | 18407 | 15134 | 14565.5 | 3133 |
| HX3 | 15969 | 13483 | 12662.6 | 5571 |
| HX4 | 18883 | 15368 | 14938.4 | 2657 |
| HX5 | 18884 | 15385 | 14938.7 | 2656 |
| HY1 | 17674 | 14290 | 14009.4 | 3866 |
| HY2 | 19040 | 15206 | 15077.8 | 2500 |
| HY3 | 18973 | 15082 | 15036.5 | 2567 |
| HY4 | 18276 | 14859 | 14485.6 | 3264 |
| JC1 | 17660 | 14462 | 14009.1 | 3880 |
| JC2 | 18537 | 15095 | 14694.2 | 3003 |
| JC3 | 18251 | 14784 | 14473.5 | 3289 |
| JC4 | 18325 | 14935 | 14512.6 | 3215 |
| JC5 | 17999 | 14711 | 14269.6 | 3541 |
| JH1 | 17937 | 14100 | 14212 | 3603 |
| JH2 | 17834 | 14071 | 14135 | 3706 |
| JH3 | 16916 | 13538 | 13418.2 | 4624 |
| JH4 | 16565 | 13220 | 13162.4 | 4975 |
| JH5 | 16886 | 13537 | 13380.4 | 4654 |
| JL1 | 17764 | 13826 | 14074.9 | 3776 |
| JL2 | 18303 | 14242 | 14497.7 | 3237 |
| JL3 | 16456 | 12993 | 13052.9 | 5084 |
| JL4 | 18352 | 14230 | 14529.7 | 3188 |
| JL5 | 17164 | 13316 | 13599.8 | 4376 |
| JW1 | 17036 | 13291 | 13508.7 | 4504 |
| JW2 | 19087 | 15231 | 15124.4 | 2453 |
| JW3 | 16351 | 12761 | 12967.5 | 5189 |
| MF1 | 17911 | 14116 | 14193.1 | 3629 |
| MF2 | 17478 | 13210 | 13854.8 | 4062 |
| MF3 | 17868 | 13773 | 14159.2 | 3672 |
| MF4 | 17926 | 14228 | 14204.3 | 3614 |
| MF5 | 17756 | 13976 | 14089.6 | 3784 |
| QHD1 | 18080 | 14544 | 14332.8 | 3460 |
| QHD2 | 18456 | 14831 | 14636.4 | 3084 |
| QHD3 | 18474 | 14702 | 14640.2 | 3066 |
| TL1 | 18678 | 14971 | 14805.9 | 2862 |
| TL2 | 18108 | 14724 | 14367.7 | 3432 |
| TL3 | 18765 | 14752 | 14874.2 | 2775 |
| TL4 | 16096 | 12628 | 12784.9 | 5444 |
| XS1 | 15516 | 11319 | 12317.9 | 6024 |
| XS2 | 16744 | 13033 | 13281.5 | 4796 |
| XS3 | 18094 | 13932 | 14329.2 | 3446 |
| XS4 | 18237 | 13970 | 14439.2 | 3303 |
| XS5 | 18060 | 13879 | 14307.5 | 3480 |
| YJP1 | 18151 | 14382 | 14393.5 | 3389 |
| YJP2 | 17471 | 13765 | 13863.3 | 4069 |
| YJP3 | 18112 | 13720 | 14356 | 3428 |
| YJP4 | 18447 | 14757 | 14631.6 | 3093 |
| YJP5 | 17950 | 14059 | 14235.1 | 3590 |
| YX1 | 18979 | 14844 | 15047.9 | 2561 |
| YX2 | 19258 | 14975 | 15257.2 | 2282 |
| YX3 | 18080 | 14272 | 14338.1 | 3460 |
| YX4 | 15772 | 12508 | 12522.6 | 5768 |
| YX5 | 17231 | 13713 | 13677.6 | 4309 |
| YX6 | 18037 | 14375 | 14316.2 | 3503 |
| YX7 | 18037 | 14443 | 14316.8 | 3503 |

Table S3 Pairwise *F*_ST_ Values among 16 Populations of *M. dioica* based on 22,868 SNPs. All P-values were significant (P< 0.01).

|  | BA | FZ | GSJ | HH | HX | HY | JC | JH | JL | JW | MF | QHD | TL | XS | YJP | YX |
| --- | --- | --- | --- | --- | --- | --- | --- | --- | --- | --- | --- | --- | --- | --- | --- | --- |
| BA | 0.0000 |  |  |  |  |  |  |  |  |  |  |  |  |  |  |  |
| FZ | 0.1484 | 0.0000 |  |  |  |  |  |  |  |  |  |  |  |  |  |  |
| GSJ | 0.1194 | 0.1650 | 0.0000 |  |  |  |  |  |  |  |  |  |  |  |  |  |
| HH | 0.2520 | 0.1517 | 0.2459 | 0.0000 |  |  |  |  |  |  |  |  |  |  |  |  |
| HX | 0.2063 | 0.2197 | 0.1992 | 0.3385 | 0.0000 |  |  |  |  |  |  |  |  |  |  |  |
| HY | 0.1836 | 0.0807 | 0.1945 | 0.1823 | 0.2653 | 0.0000 |  |  |  |  |  |  |  |  |  |  |
| JC | 0.1809 | 0.0857 | 0.1917 | 0.1944 | 0.2600 | 0.1092 | 0.0000 |  |  |  |  |  |  |  |  |  |
| JH | 0.2080 | 0.1344 | 0.2159 | 0.1257 | 0.2872 | 0.1545 | 0.1681 | 0.0000 |  |  |  |  |  |  |  |  |
| JL | 0.2012 | 0.1247 | 0.2127 | 0.1644 | 0.2860 | 0.1387 | 0.1482 | 0.1494 | 0.0000 |  |  |  |  |  |  |  |
| JW | 0.2126 | 0.0955 | 0.2137 | 0.2200 | 0.3003 | 0.1258 | 0.1118 | 0.1830 | 0.1555 | 0.0000 |  |  |  |  |  |  |
| MF | 0.1807 | 0.1094 | 0.1945 | 0.1452 | 0.2586 | 0.1204 | 0.1287 | 0.1318 | 0.0985 | 0.1397 | 0.0000 |  |  |  |  |  |
| QHD | 0.2335 | 0.1372 | 0.2339 | 0.1739 | 0.3205 | 0.1672 | 0.1727 | 0.1398 | 0.1456 | 0.1969 | 0.1299 | 0.0000 |  |  |  |  |
| TL | 0.1869 | 0.0866 | 0.1922 | 0.1942 | 0.2645 | 0.1083 | 0.0966 | 0.1674 | 0.1447 | 0.1143 | 0.1262 | 0.1726 | 0.0000 |  |  |  |
| XS | 0.2143 | 0.1334 | 0.2279 | 0.1821 | 0.3026 | 0.1507 | 0.1567 | 0.1630 | 0.1353 | 0.1744 | 0.1152 | 0.1600 | 0.1547 | 0.0000 |  |  |
| YJP | 0.1671 | 0.0894 | 0.1767 | 0.1500 | 0.2428 | 0.1048 | 0.1100 | 0.1304 | 0.1193 | 0.1182 | 0.1010 | 0.1358 | 0.1064 | 0.1298 | 0.0000 |  |
| YX | 0.1560 | 0.0866 | 0.1735 | 0.1501 | 0.2301 | 0.0963 | 0.1015 | 0.1392 | 0.1246 | 0.1082 | 0.1073 | 0.1380 | 0.0984 | 0.1383 | 0.0888 | 0.0000 |

Table S4 DIYABC parameter estimation for all the best-fitting scenarios of divergence and demography model. The q025 and q975 referred to upper and lower limit of the 95% confidence interval value.

| Divergence model (scenarios 3; *p*=0.805 [95% CI: 0.579-1.000]) | | | | |
| --- | --- | --- | --- | --- |
| Parameter | Priors | Median | q025 | q975 |
| N(group S) | 10-5.00×10^4^ | 4.14×10^4^ | 7.60×10^3^ | 4.97×10^4^ |
| N(group N) | 10-1.00×10^5^ | 8.63×10^4^ | 6.28×10^4^ | 9.94×10^4^ |
| *t*1 | 4.50×10^5^-1.50×10^6^ | 4.74×10^5^ | 4.50×10^5^ | 8.21×10^5^ |
| Demography model | | | | |
| Group N (scenarios 4; *p*=0.947 [95% CI: 0.930-0.966]) | | | | |
| NA | 10-3.00×10^4^ | 8.76e+003 | 3.13e+002 | 2.79e+004 |
| *t*2 | 100-2.00×10^6^ | 1.24e+005 | 2.75e+004 | 9.07e+005 |
| N2 | 10-2.80×10^5^ | 2.45e+005 | 1.38e+005 | 2.79e+005 |
| *t*1 | 100-2.00×10^4^ | 6.68e+003 | 5.82e+002 | 1.88e+004 |
| N1 | 10-1.50×10^5^ | 4.16e+004 | 8.34e+003 | 1.37e+005 |
| Group S (scenarios 4; *p*=0.990 [95% CI: 0.984-1.000]) | | | | |
| NA | 10-1.00×10^4^ | 4.21e+003 | 2.01e+003 | 9.51e+003 |
| *t*2 | 100-6.00×10^5^ | 3.94e+005 | 2.81e+005 | 5.87e+005 |
| N2 | 10-1.20×10^5^ | 9.38e+004 | 7.20e+004 | 1.19e+005 |
| *t*1 | 100-2.00×10^4^ | 8.91e+003 | 5.88e+003 | 1.93e+004 |
| N1 | 10-8.00×10^4^ | 1.25e+004 | 8.24e+003 | 3.40e+004 |

Table S5 Results of RDA analyses for 16 populations from *M. dioica.*

| RAD axis | RDA1 | RDA2 | RDA3 | RDA4 |
| --- | --- | --- | --- | --- |
| Total proportion explained by variables | 7.97% | 4.23% | 2.76% | 1.12% |
| Clim_PC1 | -0.088 | -0.1890 | -0.0311 | 0.018 |
| Clim_PC2 | -0.066 | 0.009 | -0.084 | -0.084 |
| Longitude | 0.013 | 0.040 | -0.005 | -0.115 |
| Latitude | -0.145 | -0.131 | 0.012 | -0.063 |

Table S6 Results of variance partitioning showing the percentage of total explained variance (% explained) in SNP variations attributable to only one factor.

| RAD axis | % of Variance Explained | df | P-Value |
| --- | --- | --- | --- |
| Clim_PC1 | 5.22 | 1 | 0.001 |
| Clim_PC2 | 1.80 | 1 | 0.065 |
| Longitude | 2.35 | 1 | 0.28 |
| Latitude | 5.86 | 1 | 0.001 |

Table S7 Outliers and associated loci based on *Helianthus annuus* genome.

| Loci | Associated variables | Location | function |
| --- | --- | --- | --- |
| 1022 | ALL | within | F-box family protein |
| 1492 | ALL | Near | reduced lateral root formation |
| 1648 | ALL | within | UDP-glucosyl transferase 85A3 |
| 2511 | ALL | Near | Preprotein translocase SecA family protein |
| 3351 | ALL | within | Probable CONTAINS InterPro DOMAIN/s: Beta-Casp domain |
| 4384 | ALL | Near | DREB and EAR motif protein 3 |
| 6873 | ALL | Near | lipid transfer protein 3 |
| 7114 | ALL | within | Putative PB1 domain; AUX/IAA protein; Auxin response factor; DNA-binding pseudobarrel domain |
| 7256 | Clim_2 | within | Putative ribosomal protein L16; Ribosomal protein S3, C-terminal; Ribosomal protein L10e/L16 |
| 7833 | Clim_1 | Near | receptor-like kinase 1 |
| 8063 | ALL | Near | Actin-binding FH2 (Formin Homology) protein |
| 8996 | ALL | within | Putative protein of unknown function DUF1754, eukaryoticLinks |
| 9902 | Clim_2 | within | Probable glucose-1-phosphate adenylyltransferase small subunit, chloroplastic |
| 12061 | ALL | Near | protein of unknown function (DUF946 fanmily) |
| 13784 | ALL | Near | RNA-binding (RRM/RBD/RNP motifs) family protein |
| 14329 | ALL | Near | F-box/RNI-like superfamily protein |
| 15680 | ALL | Near | AMP-dependent synthetase and ligase family protein |
| 16503 | ALL | Near | exoribonuclease 3 |
| 19638 | ALL | Near | FLA, FRI FRIGIDA-like protein |
| 20845 | ALL | within | Probable pleiotropic drug resistance protein TUR2 |
| 20881 | ALL | Near | O-methyltransferase 1 |
| 21034 | ALL | Near | KAS III 3-ketoacyl-acyl carrier protein synthase III |
|  |  |  |  |


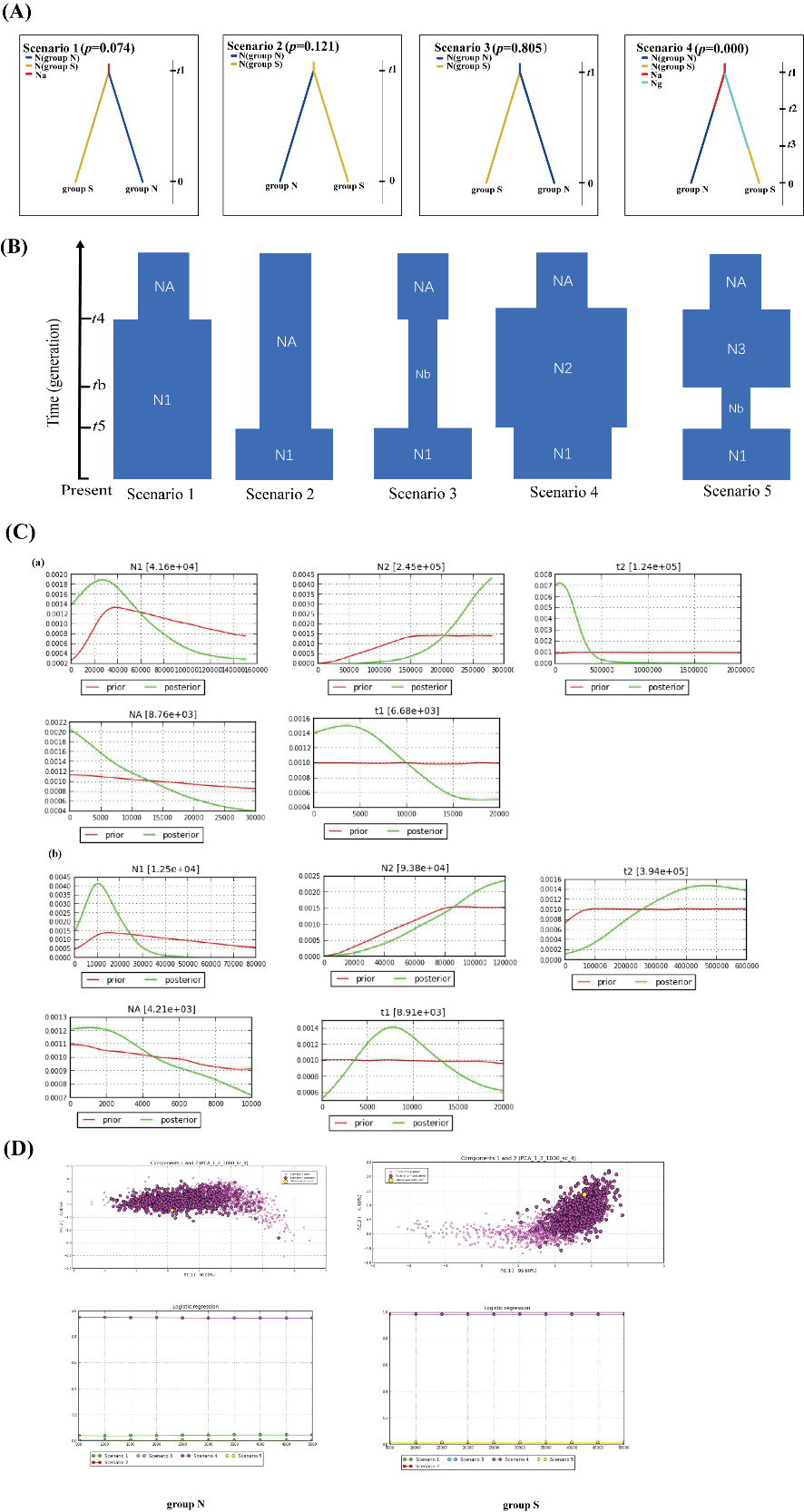


Fig. S1 (A) Divergence scenarios and corresponding posterior probabilities of *Myripnois dioica* from DIYABC. In scenario 1, group S and group N was originated form Na (ancestral population) and diverged at *t*_1_; In scenario 2, group S was set as the ancestral population, and two groups diverged at *t*_1_. In scenario 3, group N was set as the ancestral population, and two groups diverged at *t*_1_; In scenario 4, group S and group N was originated form Ng (extinct population) and Na (ancestral population), separately. Population sizes are marked in different colors. (B) Five demographic scenarios of changes in population size of *Myripnois dioica*. NA, ancestral population size; N1, current population size; N2, N3 and Nb, population sizes between NA and N1 with N2 > NA, N1>NA, N3>NA, NA>Nb, Nb<N3, N2>N1 and N1 > Nb; *t*_4_, old expansion time; *t*_b_, bottleneck time; *t*_5_, recent expansion time, *t*_5_< *t*_4_, *t*_b_< *t*_4_. (C) Prior and posterior distribution of parameters from DIY-ABC modeling of the best-fit scenarios for demographic history of group N (a) and (b) group S. (D) Plots for fitness of competing scenarios and modeling check for two groups based on direct logistic regression, simulated in DIYABC.


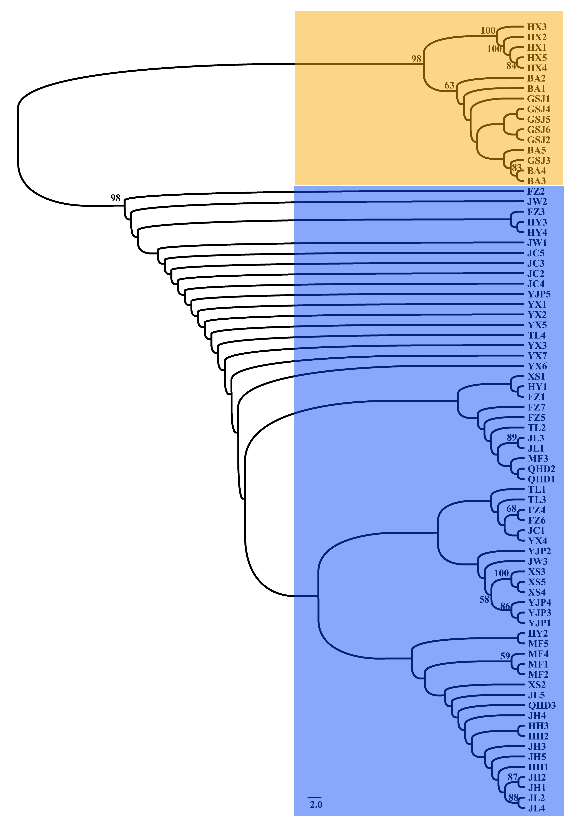


Fig. S2 Maximum Likelihood phylogeny of 77 individuals in *Myripnois dioica*. Clades with yellow and blue color are corresponding to the south and north groups.


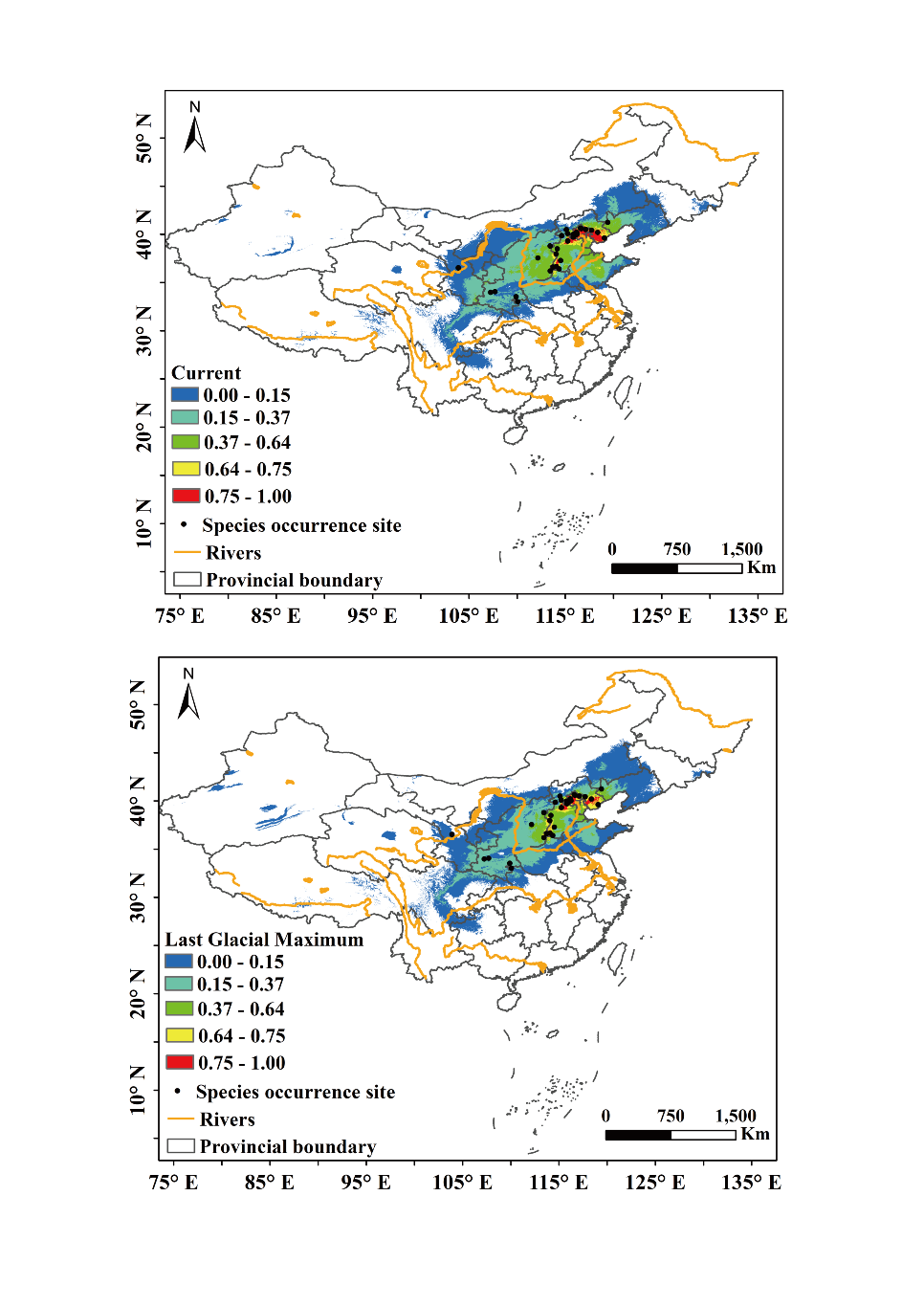


Fig. S3 Species distribution modeling of potential distribution for *Myripnois dioica* at present and during the Last Glacial Maximum *c.* 21 ka) under the CCSM model. Black dots in panel indicate the occurrence data of *Myripnois dioica*.


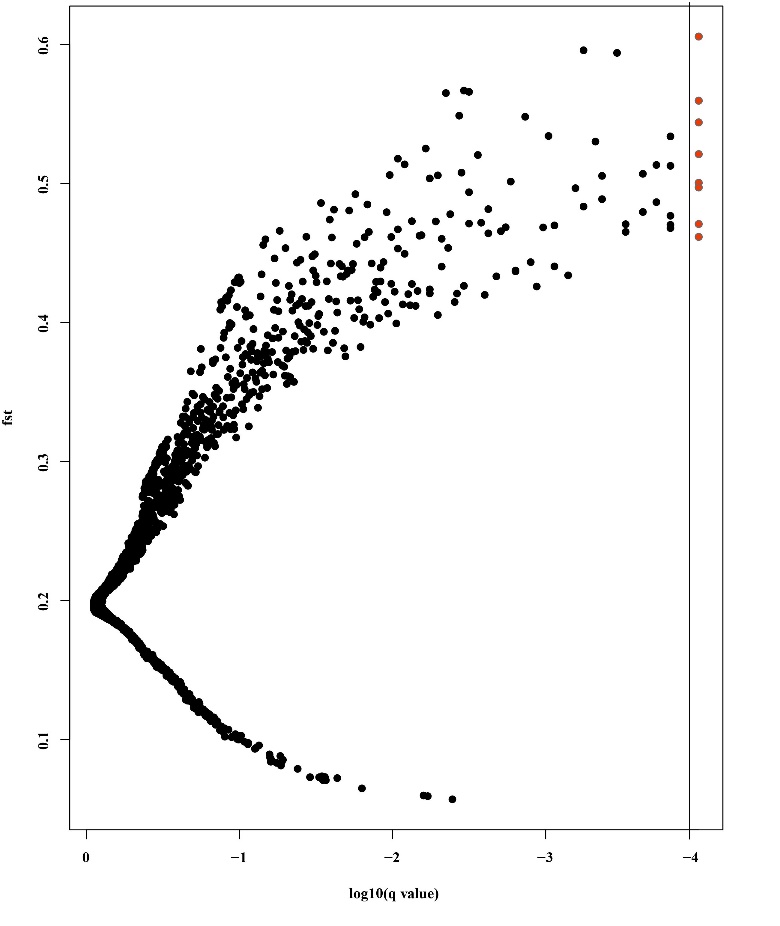


Fig. S4 The outlier loci among populations of *Myripnois dioica* by the BayeScan.


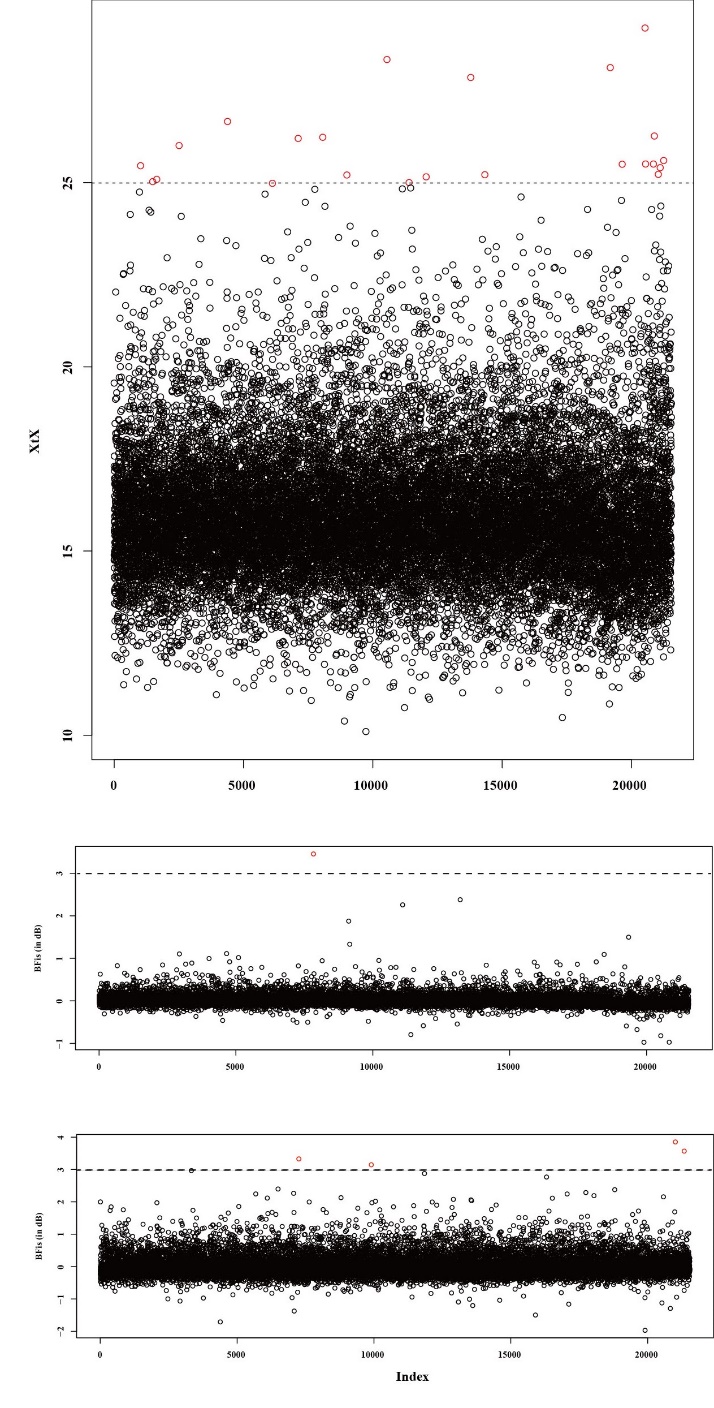


Fig. S5 The XtX-based outlier loci among all, associalted with PClim_1 and PClim_2 of *Myripnois dioica* by the BayPass. Red circles indicate the diversifying selection loci.


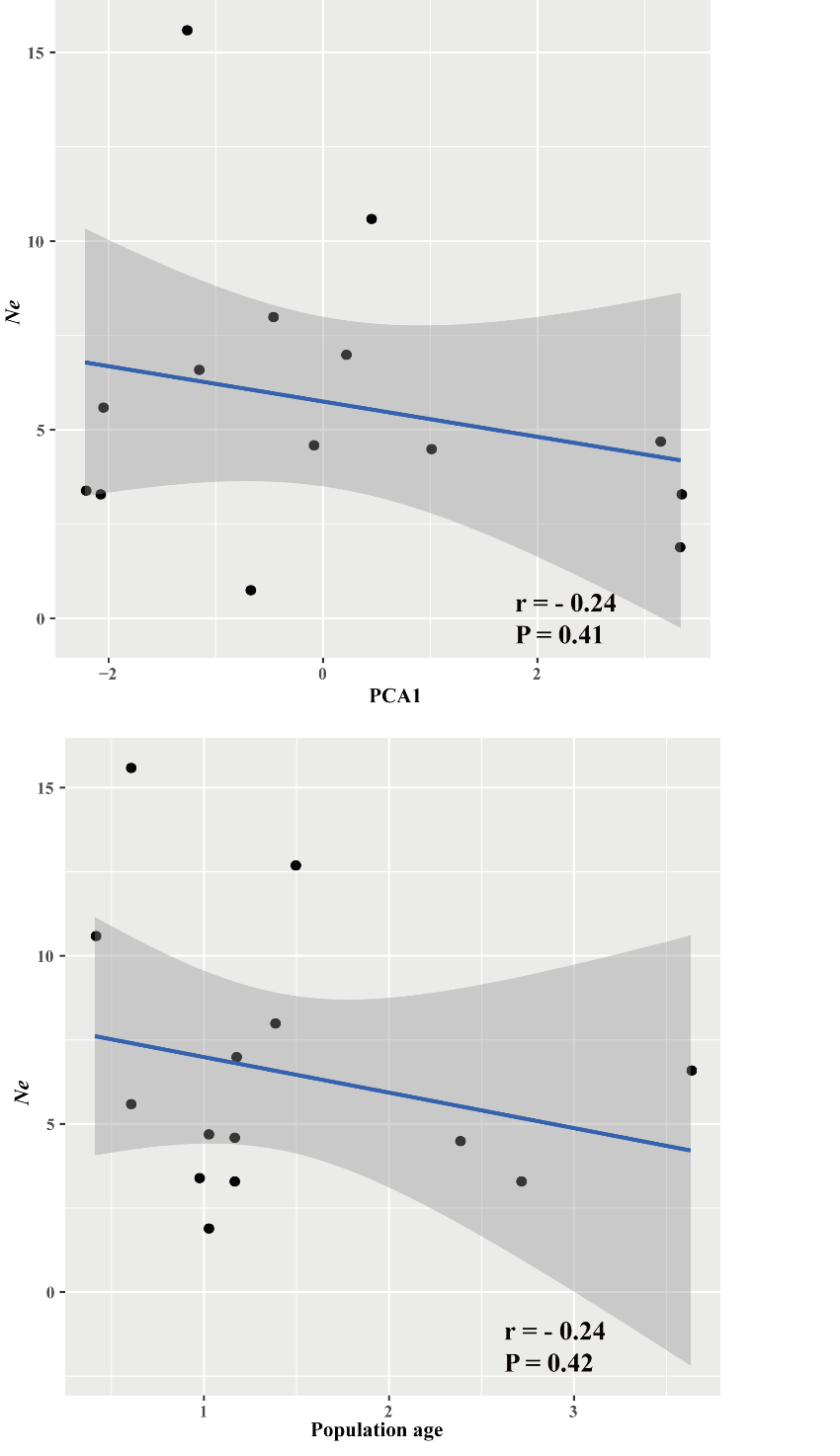


Fig. S6 Effective population size *Ne*) values are predicted by the first principal component PC1) of all the climatic variability and population age in *Myripnois dioica* populations.


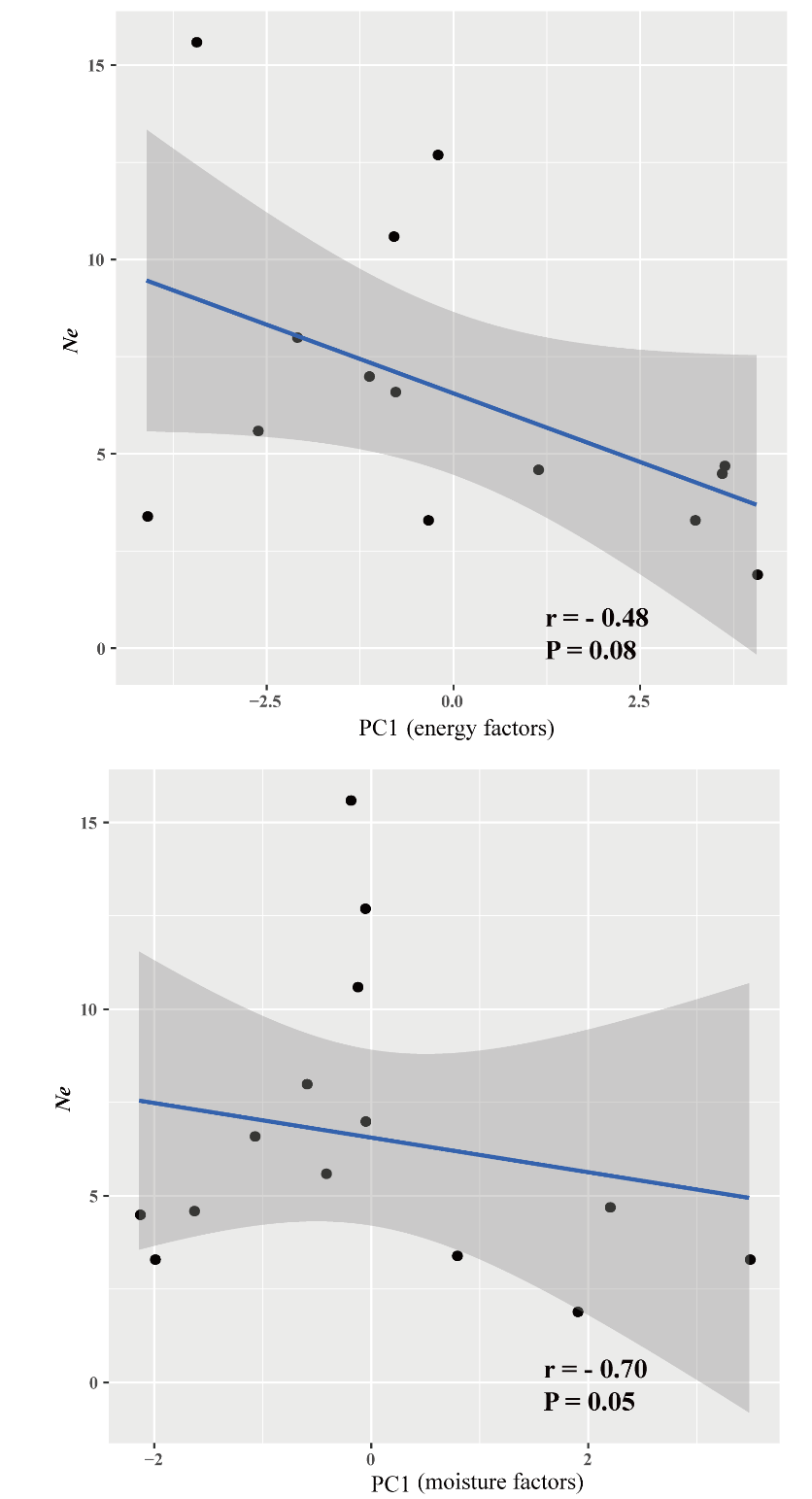


Fig. S7 Linear model between effective population size (*Ne*) among populations and climatic principal component variables (PC1) in alone energy and moisture factors.
